# Supplementary material for: Condensates of synaptic vesicles and synapsin-1 mediate actin sequestering and polymerization
Source: EMBO J. 2025 Aug 14;44(18):5112–48. doi: 10.1038/s44318-025-00516-y (PMC12436662; doi:10.1038/s44318-025-00516-y)
Supplement: Supplementary file 1 — Appendix [file 44318_2025_516_MOESM1_ESM.pdf]

APPENDIX FILE FOR:

Condensates of synaptic vesicles and synapsin are molecular beacons for actin sequestering and polymerization

Akshita Chhabra[1,2,#], Christian Hoffmann[1,2,#], Gerard Aguilar Pérez[1,2,#], Aleksandr A. Korobeinikov[1], Jakob Rentsch[3], Nadja Hümpfer[3], Linda Kokwaro[1,4], Luka Gnidovec[1], Arsen Petrovic[8], Jaqulin N. Wallace[5], Johannes Vincent Tromm[1,2,5], Cristina Román-Vendrell[5], Emma C. Johnson[5], Branislava Ranković[1,2], Eleonora Perego[6], Tommaso Volpi[7], Rubén Fernández-Busnadiego[8], Sarah Köster[6], Silvio O. Rizzoli[9], Helge Ewers[3], Jennifer R. Morgan[5], Dragomir Milovanovic[1,2,4]*

Table of Contents:

Appendix Figures S1 – page 2

Legend for Appendix Figure S1 – page 2

Appendix Figures S2 – page 3

Legend for Appendix Figure S2 – page 3

Appendix Figures S3 – page 4

Legend for Appendix Figure S3 – page 4

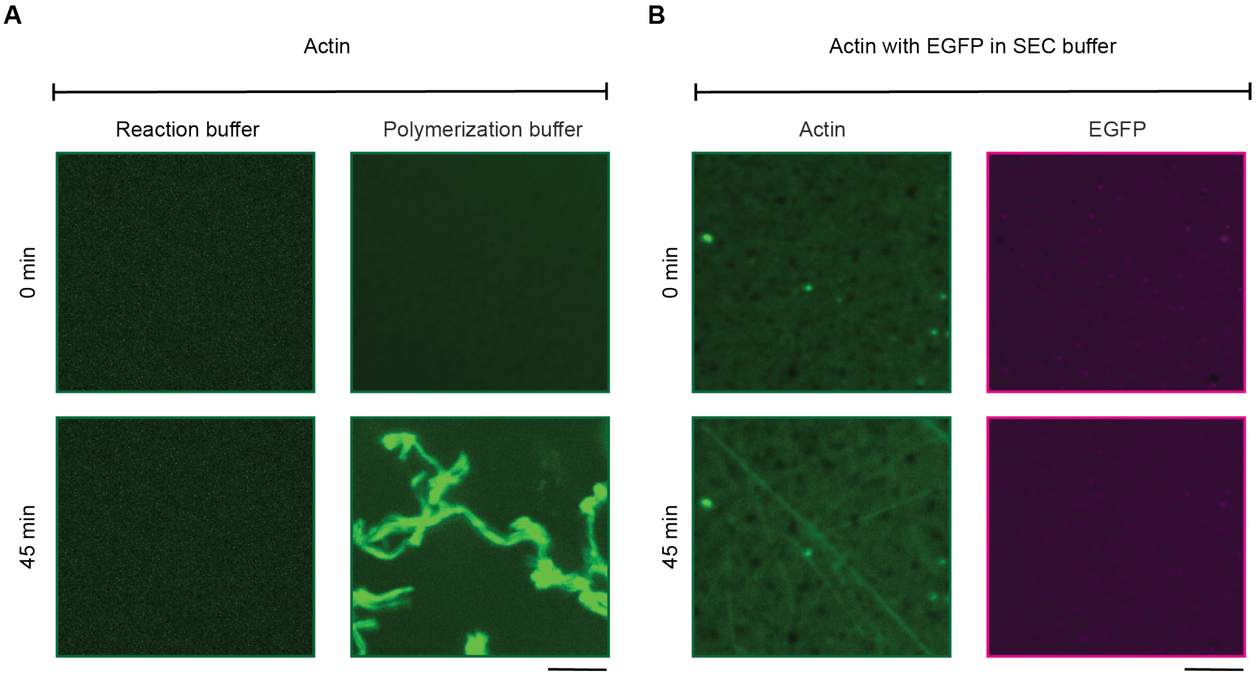

Appendix Figure S1: Positive and negative controls for actin polymerization.

A. Representative images for actin when polymerizing on its own in reaction buffer and commercial polymerization buffer (FluMaXx) at t = 0 and t = 45 min. Images were acquired using spinning-disk confocal microscope at 647 nm wavelength for actin (represented in green). Scale bar, 5 µm.

B. Representative images for actin polymerization reactions in the presence of purified EGFP and 3% PEG 8000 in reaction buffer (buffer used for final elution of the protein from the size-exclusion column, SEC buffer) at t = 0 and t = 45 min. Images were acquired using spinning-disk confocal microscope, employing the 488 nm wavelength for EGFP (control for the background signal, represented in magenta) and the 647 nm for actin (represented in green). Scale bar, 5 µm.

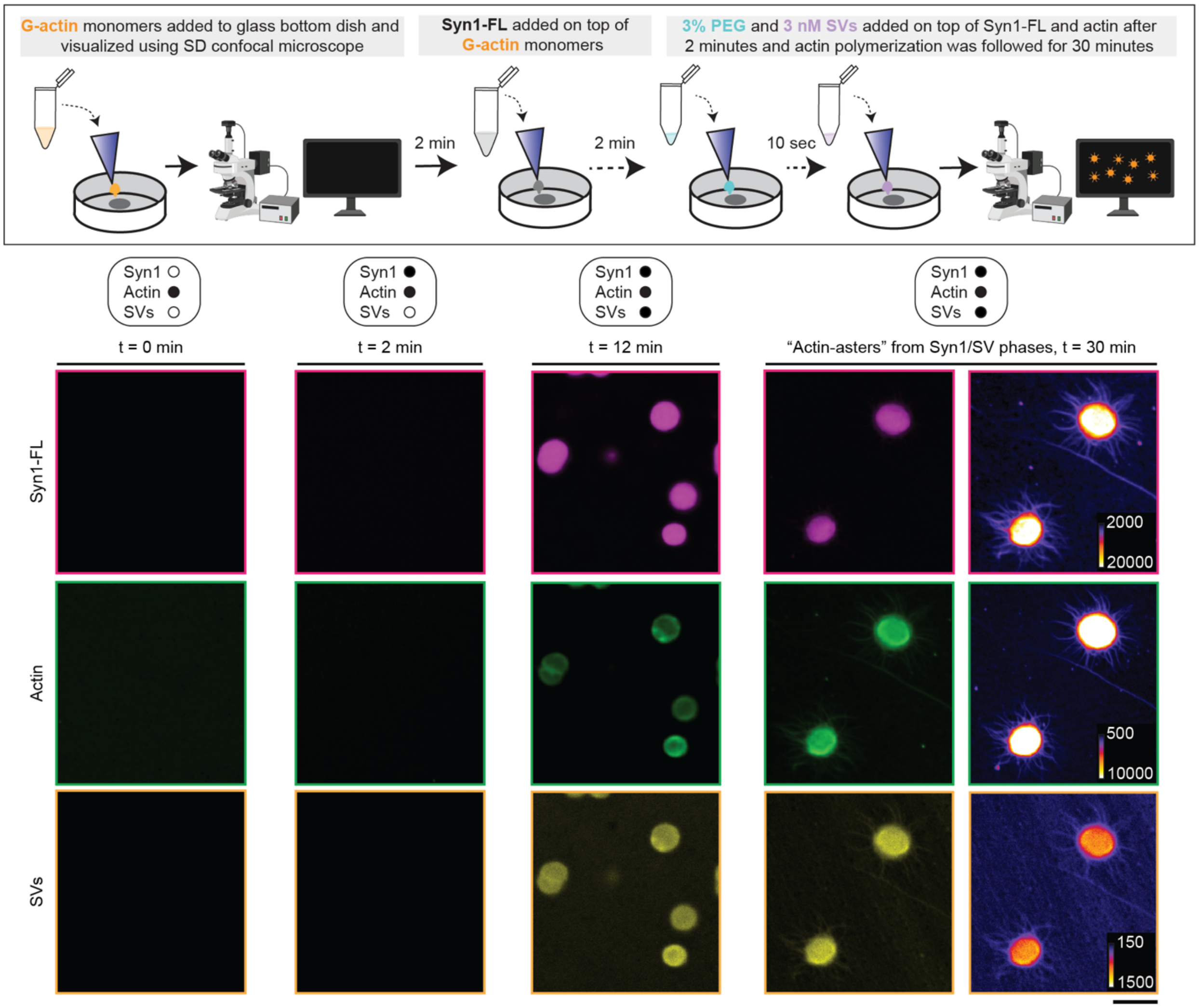

Appendix Figure S2: Actin polymerization from synapsin-1/SVs condensates.

Top: Schematic illustration showing the order of the reconstitution assay. Actin polymerization from synapsin-1/SVs condensates was assessed by first adding 4 μM ATTO647 G-actin monomers to a glass-bottom dish. Two min later, 4 μM synapsin-1 was added on top of the reaction-mix followed by addition of 3% (w/v) PEG 8,000 after 2 min. Subsequently, 3 nM SVs labeled with 1.65 μM FM4-64 dye were added to pre-formed liquid phases and actin polymerization was followed using SD confocal microscope.

Bottom: Representative SD confocal microscopy images from the reconstitution of actin with synapsin-1 and SVs in reaction buffer at t = 0, 2, 12 and 30 min. Images were acquired at 488, 561 and 647 nm wavelengths for EGFP-synapsin-1, FM4-64 labeled SVs and actin, respectively. Scale bar, 5 μm.

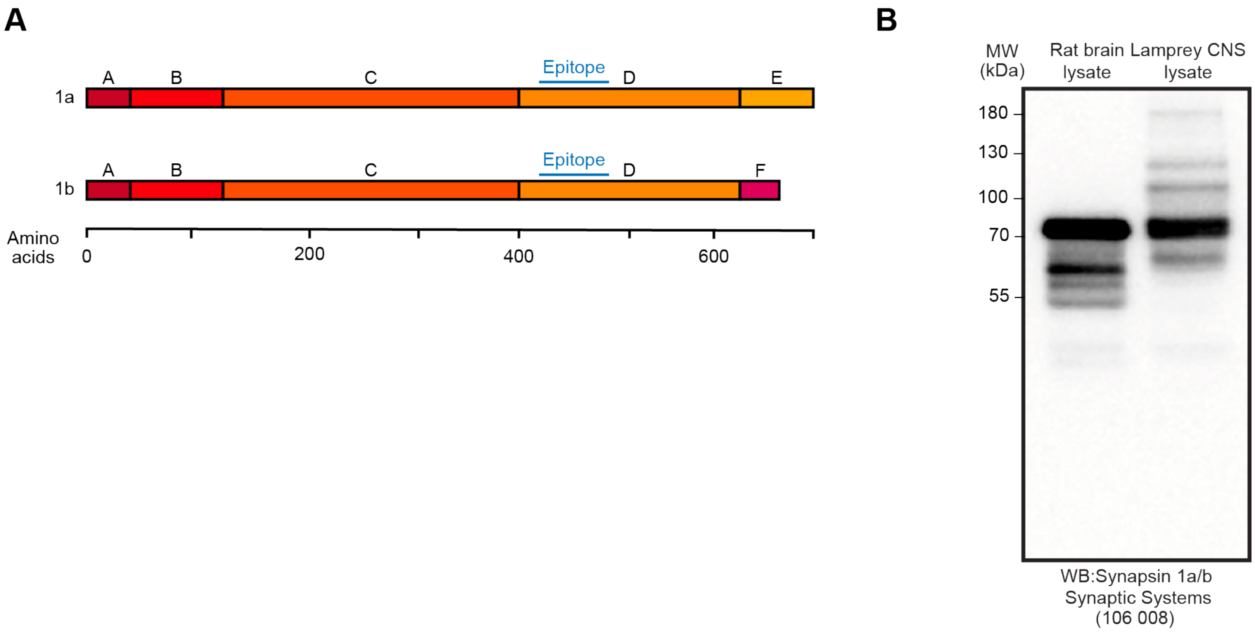

Appendix Figure S3. Characterization of antibodies against endogenous lamprey synapsin 1.
A. Scheme indicating the epitope within the regions of synapsin-1 isoforms *a* and *b*.
B. Western Blot indicating the successful recognition of synapsin 1 in both rat and lamprey CNS lysates (rabbit anti-synapsin-1 a/b antibody; Synaptic Systems 106 008).
