# Supplementary material for: Condensates of synaptic vesicles and synapsin-1 mediate actin sequestering and polymerization
Source: EMBO J. 2025 Aug 14;44(18):5112–48. doi: 10.1038/s44318-025-00516-y (PMC12436662; doi:10.1038/s44318-025-00516-y)
Supplement: Supplementary file 3 — Movie EV1 [file 44318_2025_516_MOESM3_ESM.zip › Movie EV1_Description and legend.rtf]

Movie EV1: Synapsin condensates sequester soluble G-actin and become sites of actin polymerization. Note after ~20 min the aster-like shape of polymerized actin arising from synapsin 1 full-length (Syn1-FL) condensates. Scale bar, 5 µm.
