# Supplementary material for: Condensates of synaptic vesicles and synapsin-1 mediate actin sequestering and polymerization
Source: EMBO J. 2025 Aug 14;44(18):5112–48. doi: 10.1038/s44318-025-00516-y (PMC12436662; doi:10.1038/s44318-025-00516-y)
Supplement: Supplementary file 4 — Movie EV2 [file 44318_2025_516_MOESM4_ESM.zip › Movie EV2_Description and legend.rtf]

Movie EV2: Reconstituted actin network from condensates of either synapsin 1 full-length (Syn1-FL, left) or intrinsically disordered region (Syn1-IDR, right). Scale bar, 10 µm.
