# Supplementary material for: Condensates of synaptic vesicles and synapsin-1 mediate actin sequestering and polymerization
Source: EMBO J. 2025 Aug 14;44(18):5112–48. doi: 10.1038/s44318-025-00516-y (PMC12436662; doi:10.1038/s44318-025-00516-y)
Supplement: Supplementary file 5 — Movie EV3 [file 44318_2025_516_MOESM5_ESM.zip › Movie EV3_Description and legend.rtf]

Movie EV3: 3D representation of actin networks emerging from synapsin 1 (Syn1) condensate. Note that actin fibers between asters stretch for tens of µm. Scale bar, 10 µm.
