# Supplementary material for: Condensates of synaptic vesicles and synapsin-1 mediate actin sequestering and polymerization
Source: EMBO J. 2025 Aug 14;44(18):5112–48. doi: 10.1038/s44318-025-00516-y (PMC12436662; doi:10.1038/s44318-025-00516-y)
Supplement: Supplementary file 6 — Movie EV4 [file 44318_2025_516_MOESM6_ESM.zip › Movie EV4_Description and legend.rtf]

Movie EV4: Cryo-electron tomogram of a wild-type synapse shown in Figure 7. 3D rendering of identified structures. Annotation: yellow, synaptic vesicles (SVs); green, pre-synaptic plasma membrane; blue, post-synaptic plasma membrane; red, actin filament.
