# Supplementary figures and images for: Condensates of synaptic vesicles and synapsin-1 mediate actin sequestering and polymerization (part 1 of 3)
Source: EMBO J. 2025 Aug 14;44(18):5112–48. doi: 10.1038/s44318-025-00516-y (PMC12436662; doi:10.1038/s44318-025-00516-y)

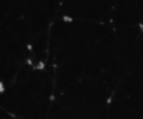

Supplement: Supplementary file 7 — Source data Fig. 1 [file 44318_2025_516_MOESM7_ESM.zip › Figure 1/Panel B/C2-Actin at 35 min.tif]

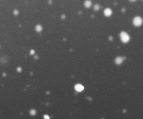

Supplement: Supplementary file 7 — Source data Fig. 1 [file 44318_2025_516_MOESM7_ESM.zip › Figure 1/Panel B/C1-Syn1_IDR at 0 min.tif]

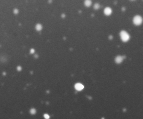

Supplement: Supplementary file 7 — Source data Fig. 1 [file 44318_2025_516_MOESM7_ESM.zip › Figure 1/Panel B/Merge_C1-Syn1_IDR and C2-Actin at 0 min.tif]

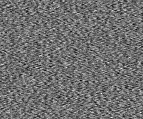

Supplement: Supplementary file 7 — Source data Fig. 1 [file 44318_2025_516_MOESM7_ESM.zip › Figure 1/Panel B/C2-Actin at 0 min.tif]

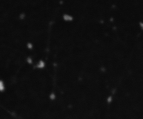

Supplement: Supplementary file 7 — Source data Fig. 1 [file 44318_2025_516_MOESM7_ESM.zip › Figure 1/Panel B/Merge_C1-Syn1_IDR and C2-Actin at 35 min.tif]

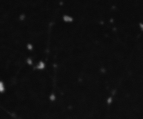

Supplement: Supplementary file 7 — Source data Fig. 1 [file 44318_2025_516_MOESM7_ESM.zip › Figure 1/Panel B/C1-Syn1_IDR at 35 min.tif]

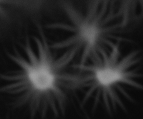

Supplement: Supplementary file 7 — Source data Fig. 1 [file 44318_2025_516_MOESM7_ESM.zip › Figure 1/Panel A/C2-Actin at 35 min.tif]

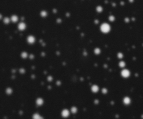

Supplement: Supplementary file 7 — Source data Fig. 1 [file 44318_2025_516_MOESM7_ESM.zip › Figure 1/Panel A/C1-Syn1 at 0 min.tif]

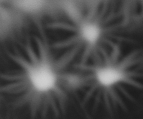

Supplement: Supplementary file 7 — Source data Fig. 1 [file 44318_2025_516_MOESM7_ESM.zip › Figure 1/Panel A/C1-Syn1 at 35 min.tif]

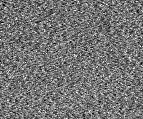

Supplement: Supplementary file 7 — Source data Fig. 1 [file 44318_2025_516_MOESM7_ESM.zip › Figure 1/Panel A/Merge_C1-Syn1 and C2-Actin at 0 min.tif]

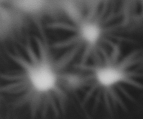

Supplement: Supplementary file 7 — Source data Fig. 1 [file 44318_2025_516_MOESM7_ESM.zip › Figure 1/Panel A/Merge_c1 and c2 at 35 min.tif]

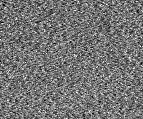

Supplement: Supplementary file 7 — Source data Fig. 1 [file 44318_2025_516_MOESM7_ESM.zip › Figure 1/Panel A/C2-Actin at 0 min.tif]

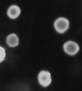

Supplement: Supplementary file 8 — Source data Fig. 2 [file 44318_2025_516_MOESM8_ESM.zip › Figure 2/Panel B/FIRE_Actin organized as rings.tif]

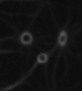

Supplement: Supplementary file 8 — Source data Fig. 2 [file 44318_2025_516_MOESM8_ESM.zip › Figure 2/Panel B/FIRE_Actin organized as Asters.tif]

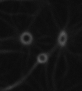

Supplement: Supplementary file 8 — Source data Fig. 2 [file 44318_2025_516_MOESM8_ESM.zip › Figure 2/Panel B/Green_Actin organized as Asters.tif]

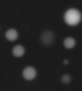

Supplement: Supplementary file 8 — Source data Fig. 2 [file 44318_2025_516_MOESM8_ESM.zip › Figure 2/Panel B/Green_Actin enriched within condensates.tif]

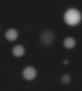

Supplement: Supplementary file 8 — Source data Fig. 2 [file 44318_2025_516_MOESM8_ESM.zip › Figure 2/Panel B/FIRE_Actin enriched within condensates.tif]

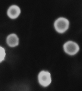

Supplement: Supplementary file 8 — Source data Fig. 2 [file 44318_2025_516_MOESM8_ESM.zip › Figure 2/Panel B/Green_Actin organized as rings.tif]

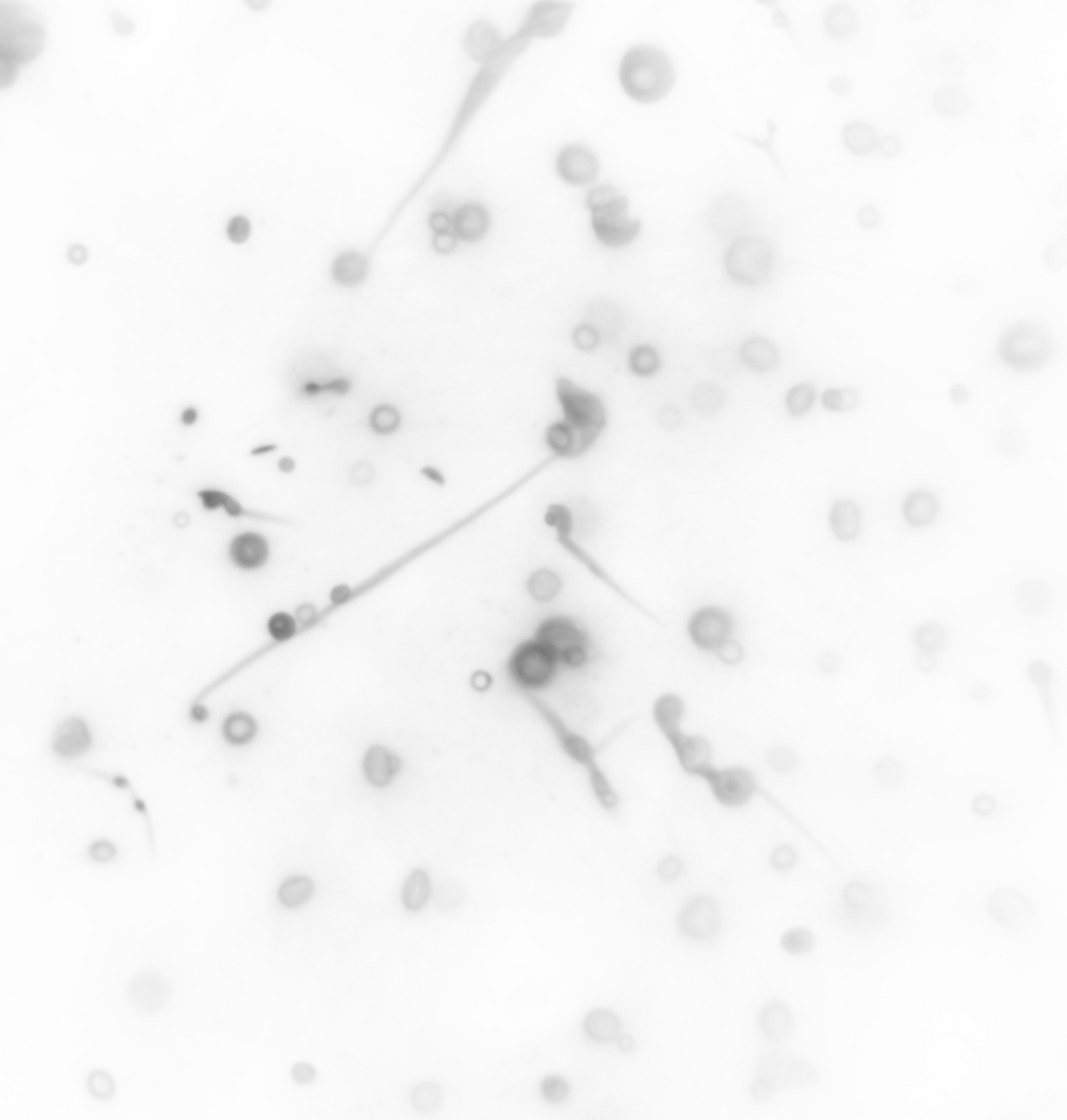

Supplement: Supplementary file 8 — Source data Fig. 2 [file 44318_2025_516_MOESM8_ESM.zip › Figure 2/Panel D/Actin after 30 min.tif]

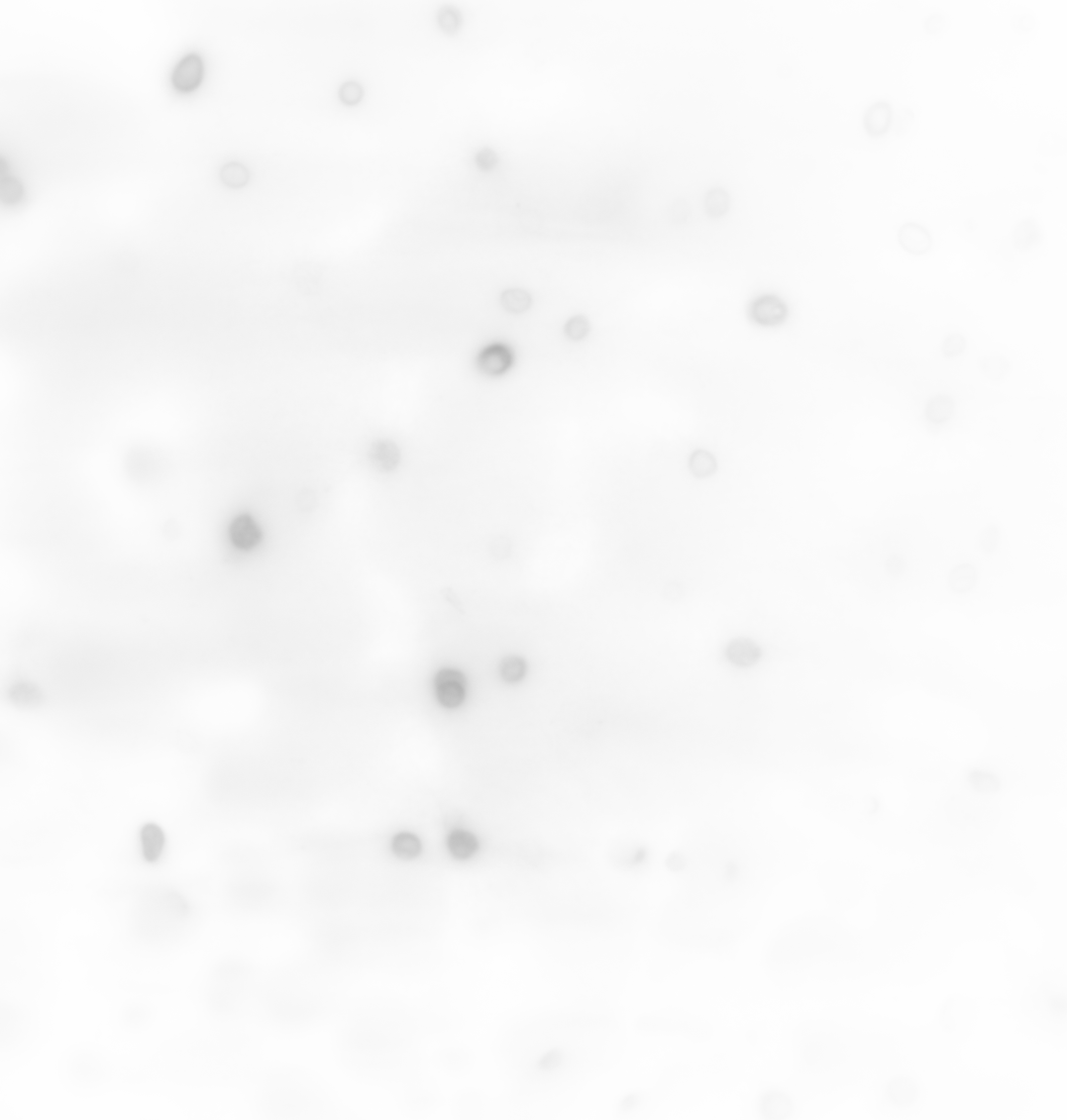

Supplement: Supplementary file 8 — Source data Fig. 2 [file 44318_2025_516_MOESM8_ESM.zip › Figure 2/Panel D/Actin before 30 min.tif]

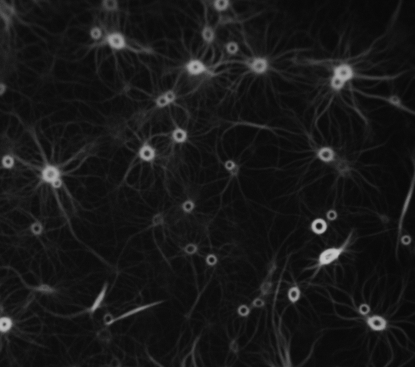

Supplement: Supplementary file 8 — Source data Fig. 2 [file 44318_2025_516_MOESM8_ESM.zip › Figure 2/Panel C/Actin asters network_FIRE.tif]

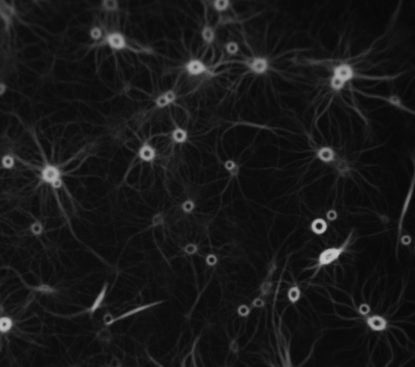

Supplement: Supplementary file 8 — Source data Fig. 2 [file 44318_2025_516_MOESM8_ESM.zip › Figure 2/Panel C/Actin asters network_Green.tif]

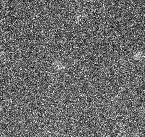

Supplement: Supplementary file 8 — Source data Fig. 2 [file 44318_2025_516_MOESM8_ESM.zip › Figure 2/Panel A/T0-min_actin.tif]

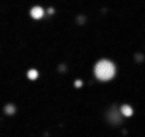

Supplement: Supplementary file 8 — Source data Fig. 2 [file 44318_2025_516_MOESM8_ESM.zip › Figure 2/Panel A/T20-min_actin.tif]

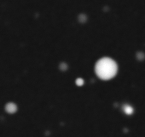

Supplement: Supplementary file 8 — Source data Fig. 2 [file 44318_2025_516_MOESM8_ESM.zip › Figure 2/Panel A/T10-min_Syn1.tif]

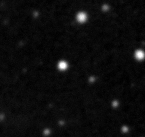

Supplement: Supplementary file 8 — Source data Fig. 2 [file 44318_2025_516_MOESM8_ESM.zip › Figure 2/Panel A/T0-min_Syn1.tif]

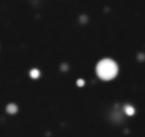

Supplement: Supplementary file 8 — Source data Fig. 2 [file 44318_2025_516_MOESM8_ESM.zip › Figure 2/Panel A/T20-min_Syn1.tif]

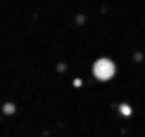

Supplement: Supplementary file 8 — Source data Fig. 2 [file 44318_2025_516_MOESM8_ESM.zip › Figure 2/Panel A/T10-min_actin.tif]

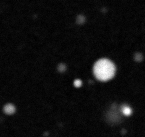

Supplement: Supplementary file 8 — Source data Fig. 2 [file 44318_2025_516_MOESM8_ESM.zip › Figure 2/Panel A/T15-min_actin.tif]

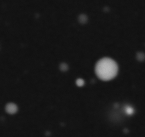

Supplement: Supplementary file 8 — Source data Fig. 2 [file 44318_2025_516_MOESM8_ESM.zip › Figure 2/Panel A/T15-min_Syn1.tif]

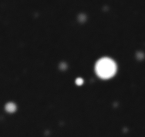

Supplement: Supplementary file 8 — Source data Fig. 2 [file 44318_2025_516_MOESM8_ESM.zip › Figure 2/Panel A/T5-min_Syn1.tif]

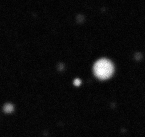

Supplement: Supplementary file 8 — Source data Fig. 2 [file 44318_2025_516_MOESM8_ESM.zip › Figure 2/Panel A/T5-min_actin.tif]

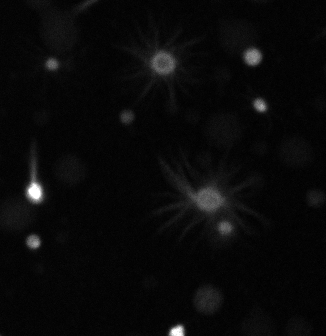

Supplement: Supplementary file 9 — Source data Fig. 3 [file 44318_2025_516_MOESM9_ESM.zip › Figure 3/Panel B/PKA_FIRE.tif]

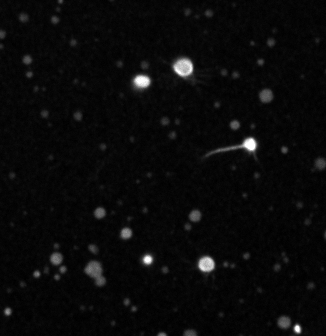

Supplement: Supplementary file 9 — Source data Fig. 3 [file 44318_2025_516_MOESM9_ESM.zip › Figure 3/Panel B/CamKII_FIRE.tif]

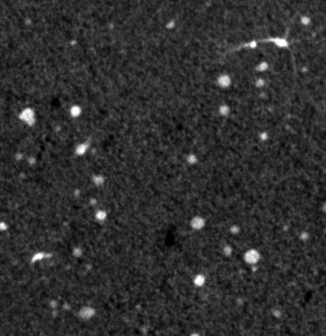

Supplement: Supplementary file 9 — Source data Fig. 3 [file 44318_2025_516_MOESM9_ESM.zip › Figure 3/Panel B/PKA-CaMKII_FIRE.tif]

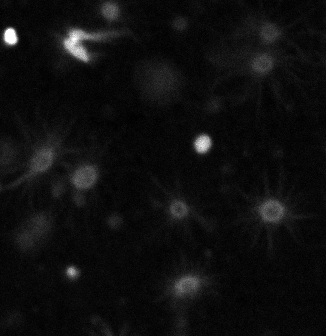

Supplement: Supplementary file 9 — Source data Fig. 3 [file 44318_2025_516_MOESM9_ESM.zip › Figure 3/Panel B/WT_FIRE.tif]

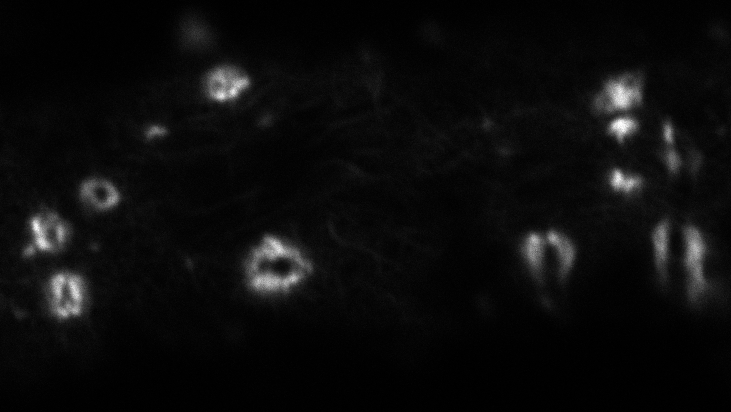

Supplement: Supplementary file 10 — Source data Fig. 4 [file 44318_2025_516_MOESM10_ESM.zip › Figure 4/Panel D/Merge.tif]

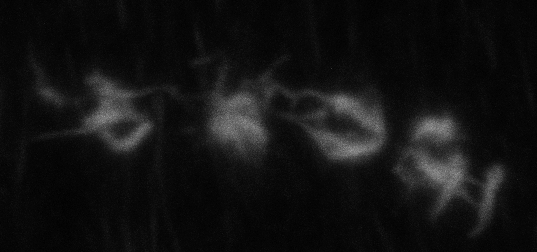

Supplement: Supplementary file 10 — Source data Fig. 4 [file 44318_2025_516_MOESM10_ESM.zip › Figure 4/Panel F/Individual synapse showing aster structures_Increased contrast.tif]

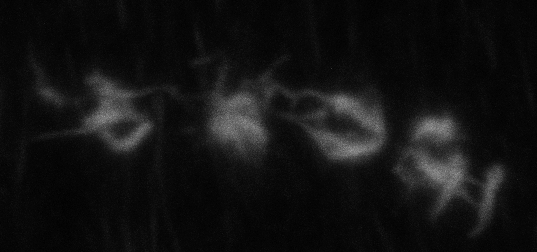

Supplement: Supplementary file 10 — Source data Fig. 4 [file 44318_2025_516_MOESM10_ESM.zip › Figure 4/Panel F/Individual synapse showing aster structures.tif]

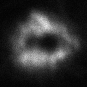

Supplement: Supplementary file 10 — Source data Fig. 4 [file 44318_2025_516_MOESM10_ESM.zip › Figure 4/Panel D/MAGNIFIED/Merge_Zoom 2.tif]

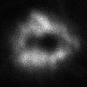

Supplement: Supplementary file 10 — Source data Fig. 4 [file 44318_2025_516_MOESM10_ESM.zip › Figure 4/Panel D/MAGNIFIED/C1-Merge_Zoom 2_ACTIN_GREEN.tif]

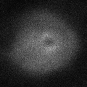

Supplement: Supplementary file 10 — Source data Fig. 4 [file 44318_2025_516_MOESM10_ESM.zip › Figure 4/Panel D/MAGNIFIED/C2-Merge_Zoom 2_SYN1_MAGENTA.tif]

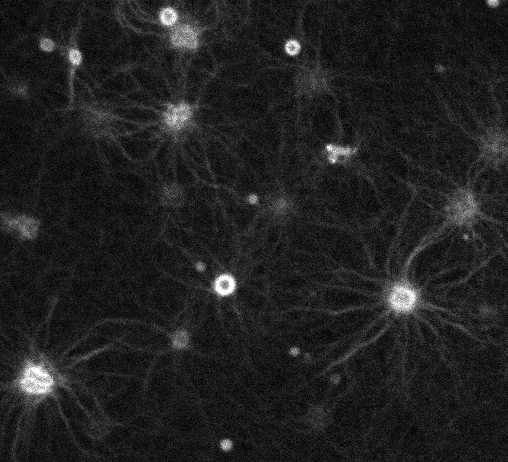

Supplement: Supplementary file 10 — Source data Fig. 4 [file 44318_2025_516_MOESM10_ESM.zip › Figure 4/Panel A/After Polymerization_35 min/SYN_SVS_actin_005_good image_ROTATED_CUTOUT.tif]

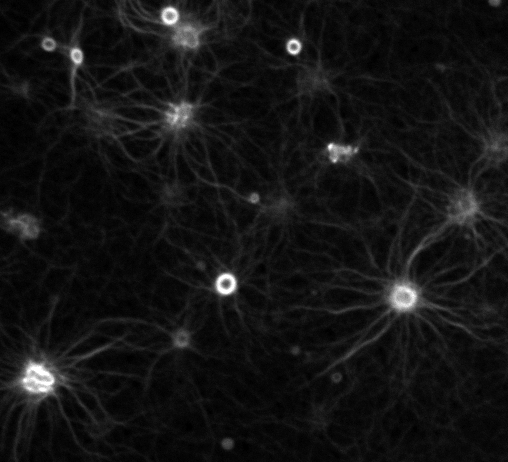

Supplement: Supplementary file 10 — Source data Fig. 4 [file 44318_2025_516_MOESM10_ESM.zip › Figure 4/Panel A/After Polymerization_35 min/C2-SYN_SVS_actin_005_good image_ROTATED_CUTOUT_SVs_ORANGE.tif]

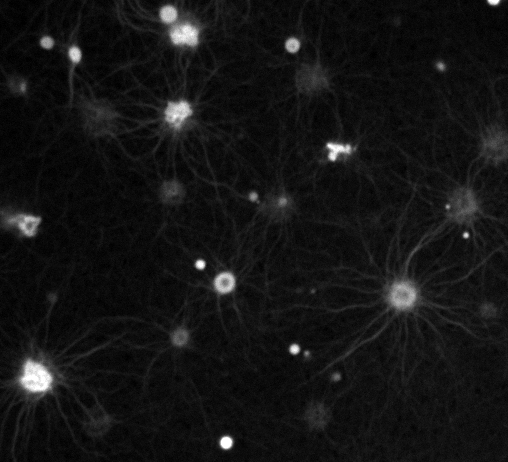

Supplement: Supplementary file 10 — Source data Fig. 4 [file 44318_2025_516_MOESM10_ESM.zip › Figure 4/Panel A/After Polymerization_35 min/C1-SYN_SVS_actin_005_good image_ROTATED_CUTOUT_SYN1_MAGENTA.tif]

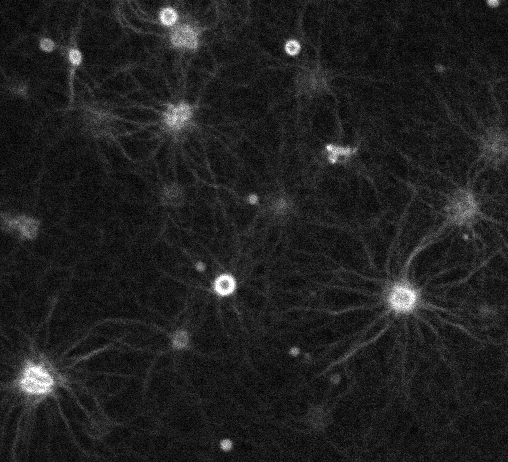

Supplement: Supplementary file 10 — Source data Fig. 4 [file 44318_2025_516_MOESM10_ESM.zip › Figure 4/Panel A/After Polymerization_35 min/C3-SYN_SVS_actin_005_good image_ROTATED_CUTOUT_ACTIN_GREEN.tif]

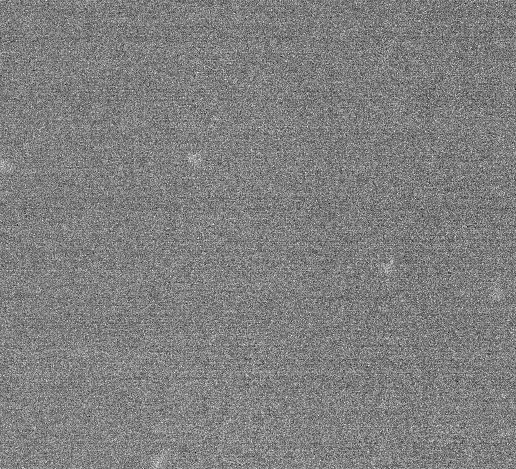

Supplement: Supplementary file 10 — Source data Fig. 4 [file 44318_2025_516_MOESM10_ESM.zip › Figure 4/Panel A/Before Polymerization_0 min/Merged_all channels.tif]

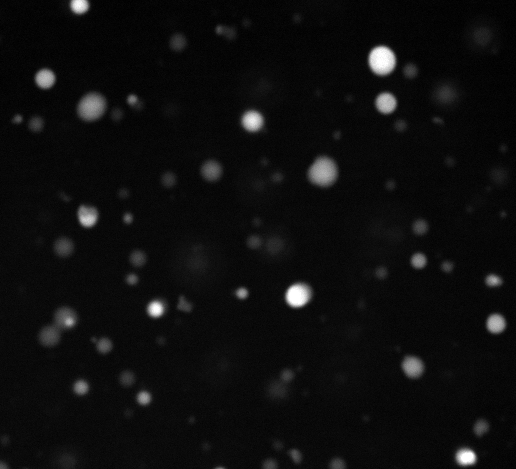

Supplement: Supplementary file 10 — Source data Fig. 4 [file 44318_2025_516_MOESM10_ESM.zip › Figure 4/Panel A/Before Polymerization_0 min/C1-Composite_ROTATED_CUTOUT_SVs_ORANGE.tif]

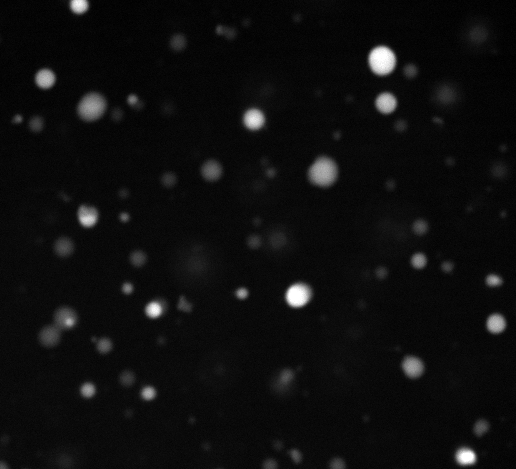

Supplement: Supplementary file 10 — Source data Fig. 4 [file 44318_2025_516_MOESM10_ESM.zip › Figure 4/Panel A/Before Polymerization_0 min/C3-Composite_ROTATED_CUTOUT_SYN1_MAGENTA.tif]

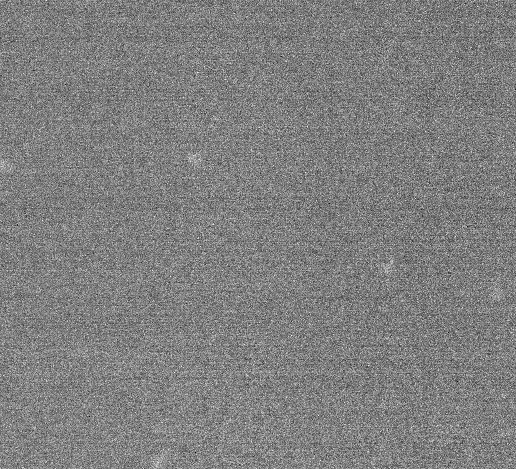

Supplement: Supplementary file 10 — Source data Fig. 4 [file 44318_2025_516_MOESM10_ESM.zip › Figure 4/Panel A/Before Polymerization_0 min/C2-Composite_ROTATED_CUTOUT_ACTIN_GREEN.tif]

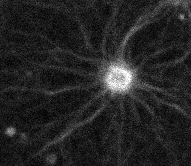

Supplement: Supplementary file 10 — Source data Fig. 4 [file 44318_2025_516_MOESM10_ESM.zip › Figure 4/Panel A/After Polymerization_35 min/MAGNIFIED/C3-SYN_SVS_actin_005_good image_ROTATED_CUTOUT_ZOOM_ACTIN_GREEN.tif]

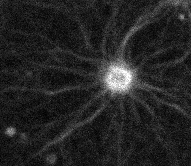

Supplement: Supplementary file 10 — Source data Fig. 4 [file 44318_2025_516_MOESM10_ESM.zip › Figure 4/Panel A/After Polymerization_35 min/MAGNIFIED/SYN_SVS_actin_005_good image_ROTATED_CUTOUT_ZOOM.tif]

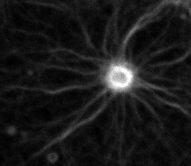

Supplement: Supplementary file 10 — Source data Fig. 4 [file 44318_2025_516_MOESM10_ESM.zip › Figure 4/Panel A/After Polymerization_35 min/MAGNIFIED/C2-SYN_SVS_actin_005_good image_ROTATED_CUTOUT_ZOOM_SVs_ORANGE.tif]

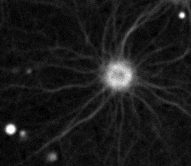

Supplement: Supplementary file 10 — Source data Fig. 4 [file 44318_2025_516_MOESM10_ESM.zip › Figure 4/Panel A/After Polymerization_35 min/MAGNIFIED/C1-SYN_SVS_actin_005_good image_ROTATED_CUTOUT_ZOOM_SYN1_MAGENTA.tif]

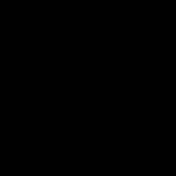

Supplement: Supplementary file 11 — Source data Fig. 5 [file 44318_2025_516_MOESM11_ESM.zip › Figure 5/Panel B/2 min/C2-Syn1 at 2 min.tif]

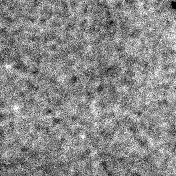

Supplement: Supplementary file 11 — Source data Fig. 5 [file 44318_2025_516_MOESM11_ESM.zip › Figure 5/Panel B/2 min/C1-Actin at 2 min.tif]

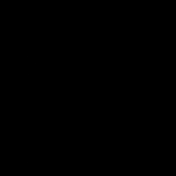

Supplement: Supplementary file 11 — Source data Fig. 5 [file 44318_2025_516_MOESM11_ESM.zip › Figure 5/Panel B/0 min/Synapsin 1 at 0 min.tif]

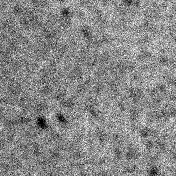

Supplement: Supplementary file 11 — Source data Fig. 5 [file 44318_2025_516_MOESM11_ESM.zip › Figure 5/Panel B/0 min/Actin at 0 min.tif]

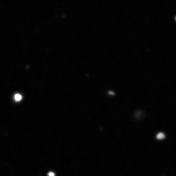

Supplement: Supplementary file 11 — Source data Fig. 5 [file 44318_2025_516_MOESM11_ESM.zip › Figure 5/Panel B/30 min/C2-Syn1 at 30 min.tif]

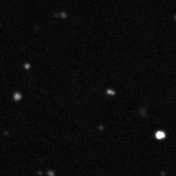

Supplement: Supplementary file 11 — Source data Fig. 5 [file 44318_2025_516_MOESM11_ESM.zip › Figure 5/Panel B/30 min/C1-Actin at 30 min.tif]

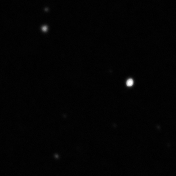

Supplement: Supplementary file 11 — Source data Fig. 5 [file 44318_2025_516_MOESM11_ESM.zip › Figure 5/Panel B/15 min/C1-Actin at 15 min.tif]

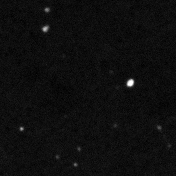

Supplement: Supplementary file 11 — Source data Fig. 5 [file 44318_2025_516_MOESM11_ESM.zip › Figure 5/Panel B/15 min/C2-Syn1 at 15 min.tif]

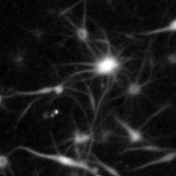

Supplement: Supplementary file 11 — Source data Fig. 5 [file 44318_2025_516_MOESM11_ESM.zip › Figure 5/Panel D/0 min/C2-Syn1 at 0 min_FIRE.tif]

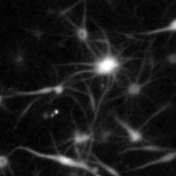

Supplement: Supplementary file 11 — Source data Fig. 5 [file 44318_2025_516_MOESM11_ESM.zip › Figure 5/Panel D/0 min/C2-Syn1 at 0 min_Magenta.tif]

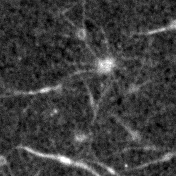

Supplement: Supplementary file 11 — Source data Fig. 5 [file 44318_2025_516_MOESM11_ESM.zip › Figure 5/Panel D/0 min/C1-Actin at 0 min_Green.tif]

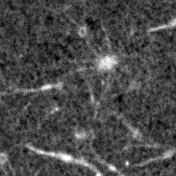

Supplement: Supplementary file 11 — Source data Fig. 5 [file 44318_2025_516_MOESM11_ESM.zip › Figure 5/Panel D/30 min/C1-Actin at 30 min_Green.tif]

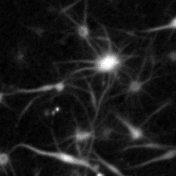

Supplement: Supplementary file 11 — Source data Fig. 5 [file 44318_2025_516_MOESM11_ESM.zip › Figure 5/Panel D/30 min/C2-Syn1 at 30 min_Magenta.tif]

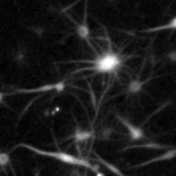

Supplement: Supplementary file 11 — Source data Fig. 5 [file 44318_2025_516_MOESM11_ESM.zip › Figure 5/Panel D/30 min/C2-Syn1 at 30 min_FIRE.tif]

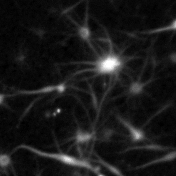

Supplement: Supplementary file 11 — Source data Fig. 5 [file 44318_2025_516_MOESM11_ESM.zip › Figure 5/Panel D/15 min/C2-Syn1 at 15 min_Magenta.tif]

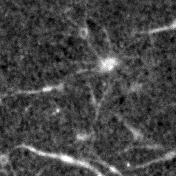

Supplement: Supplementary file 11 — Source data Fig. 5 [file 44318_2025_516_MOESM11_ESM.zip › Figure 5/Panel D/15 min/C1-Actin at 15 min_Green.tif]

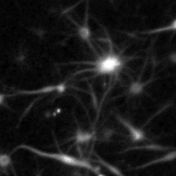

Supplement: Supplementary file 11 — Source data Fig. 5 [file 44318_2025_516_MOESM11_ESM.zip › Figure 5/Panel D/15 min/C2-Syn1 at 15 min_FIRE.tif]

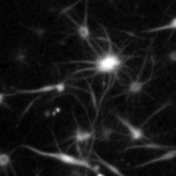

Supplement: Supplementary file 11 — Source data Fig. 5 [file 44318_2025_516_MOESM11_ESM.zip › Figure 5/Panel D/Before Lat A/C2-Syn1 at before-Lat-A_FIRE.tif]

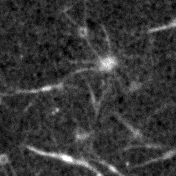

Supplement: Supplementary file 11 — Source data Fig. 5 [file 44318_2025_516_MOESM11_ESM.zip › Figure 5/Panel D/Before Lat A/C1-Actin at before-Lat-A_Green.tif]

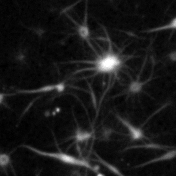

Supplement: Supplementary file 11 — Source data Fig. 5 [file 44318_2025_516_MOESM11_ESM.zip › Figure 5/Panel D/Before Lat A/C2-Syn1 at before-Lat-A_Magenta.tif]

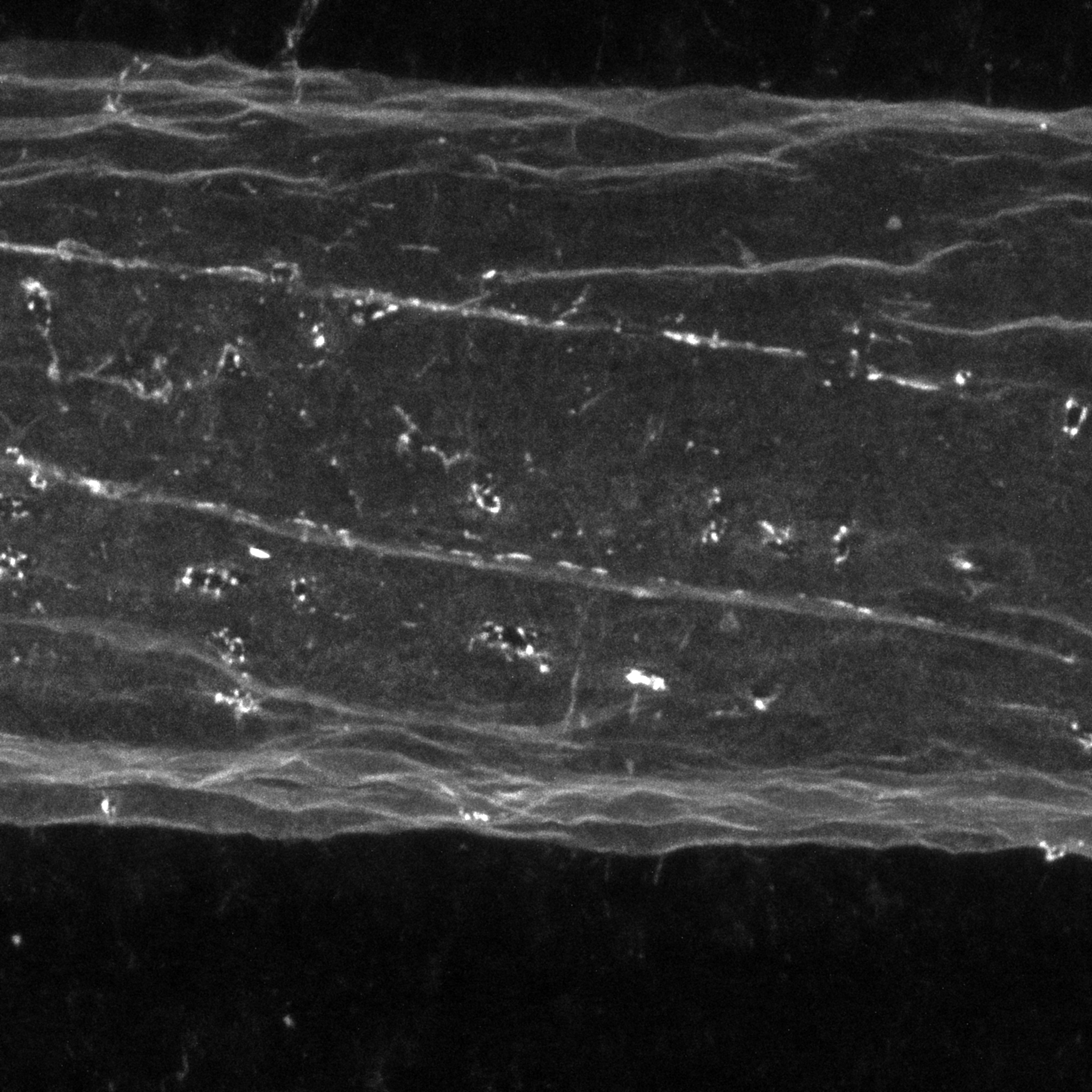

Supplement: Supplementary file 11 — Source data Fig. 5 [file 44318_2025_516_MOESM11_ESM.zip › Figure 5/Panel F/Lat A/MAX_082524_Prep3A_LatA1+-PhalloidinA488_Axon2_Section002_ch00-z1-49.tif]

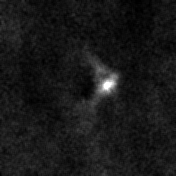

Supplement: Supplementary file 11 — Source data Fig. 5 [file 44318_2025_516_MOESM11_ESM.zip › Figure 5/Panel F/Lat A/082524_Prep3A_LatA1+-PhalloidinA488_Axon2_Section002_ch00-z3-crop1.tif]

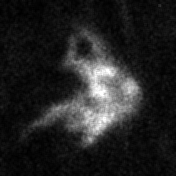

Supplement: Supplementary file 11 — Source data Fig. 5 [file 44318_2025_516_MOESM11_ESM.zip › Figure 5/Panel F/Control/082424_Prep2A_0.03%DMSO-phalloidinA88_Axon2_Section-004_ch00-z5-crop1.tif]

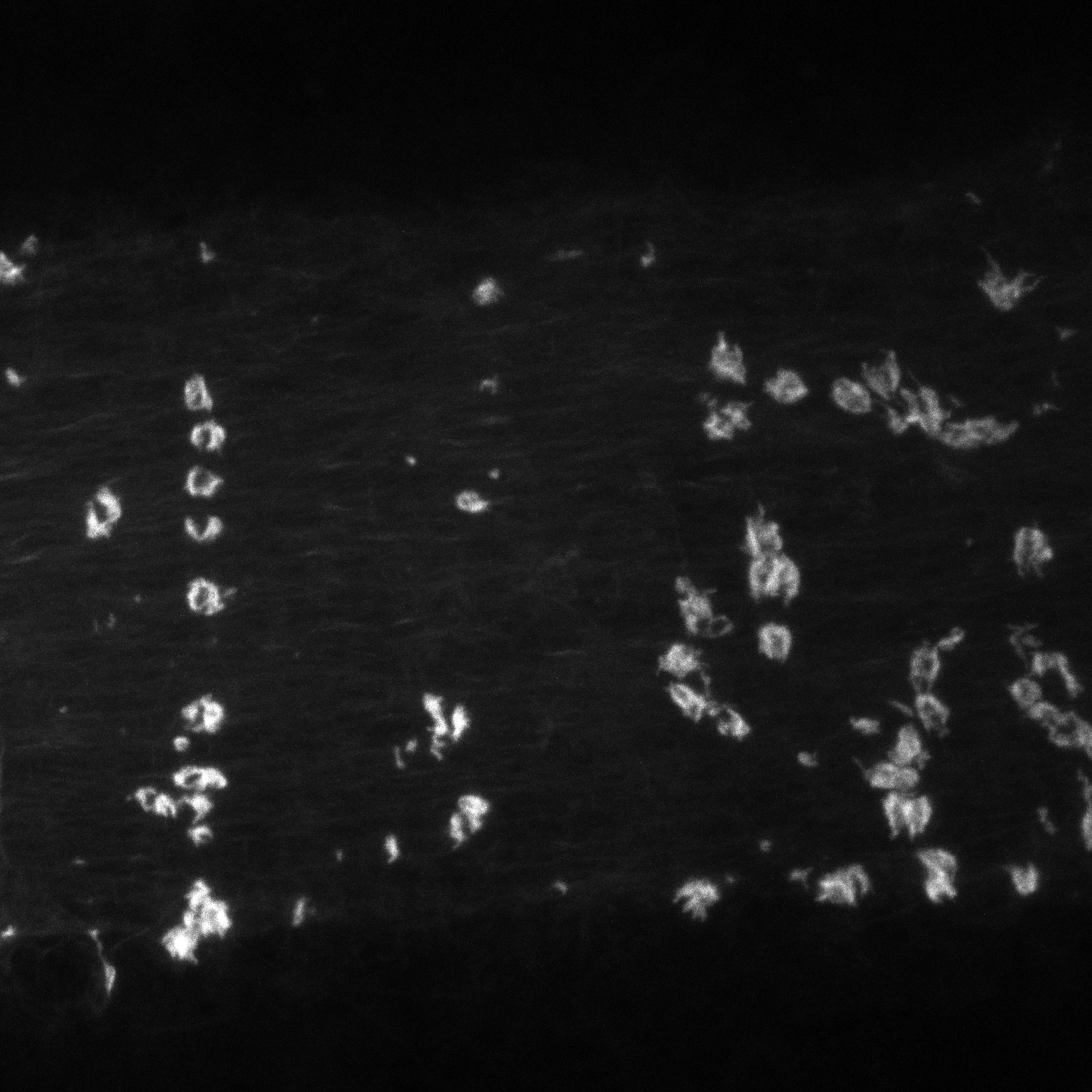

Supplement: Supplementary file 11 — Source data Fig. 5 [file 44318_2025_516_MOESM11_ESM.zip › Figure 5/Panel F/Control/MAX_082424_Prep2A_0.03%DMSO-phalloidinA88_Axon2_Section-004_ch00-z3-21.tif]

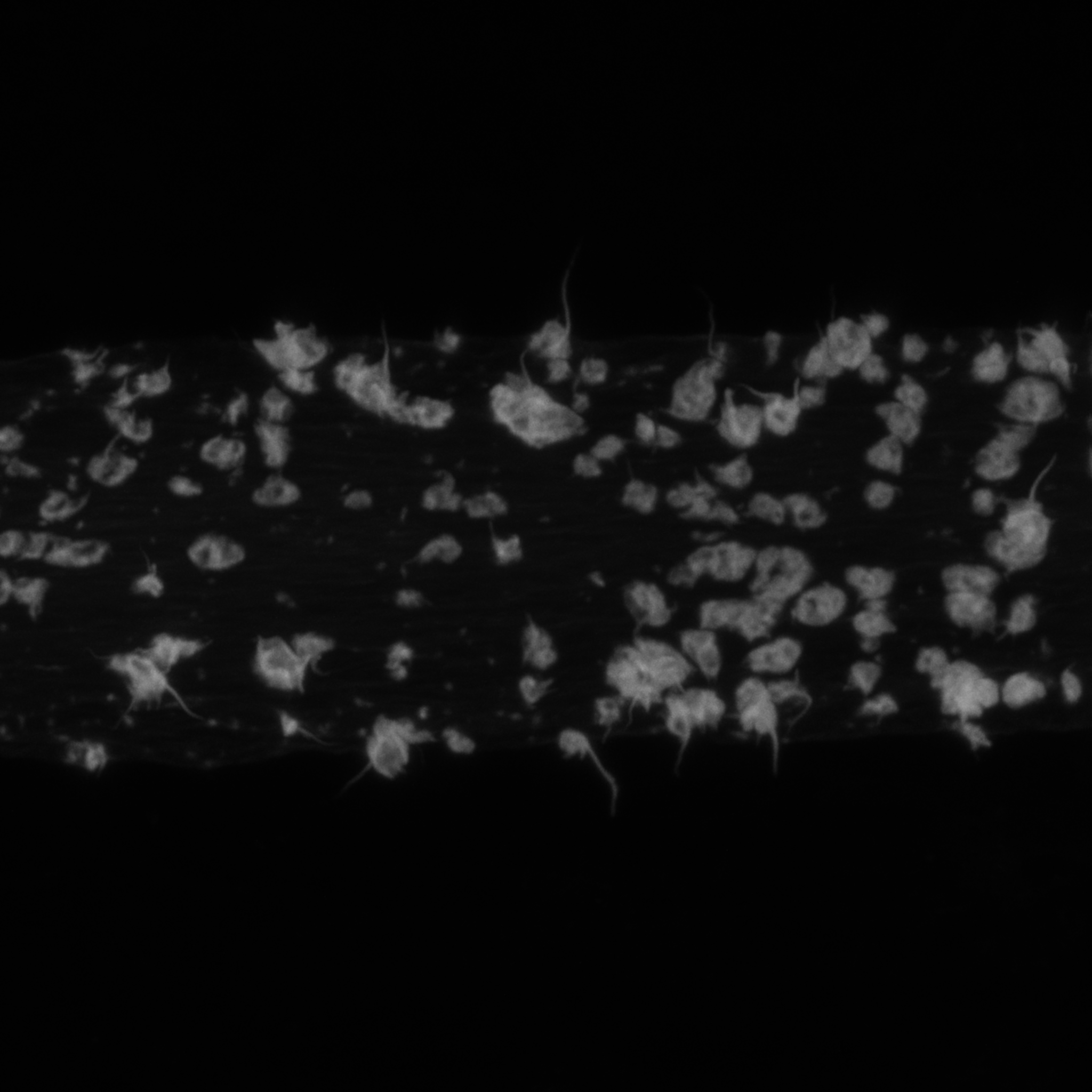

Supplement: Supplementary file 11 — Source data Fig. 5 [file 44318_2025_516_MOESM11_ESM.zip › Figure 5/Panel G/Lat A/MAX_082524_Prep2C_phalloidinA488-LatA2+_Axon1_Section-003_ch00-z4-40.tif]

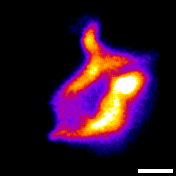

Supplement: Supplementary file 11 — Source data Fig. 5 [file 44318_2025_516_MOESM11_ESM.zip › Figure 5/Panel G/Lat A/082524_Prep2C_phalloidinA488-LatA2+_Axon1_Section-003_ch00-z4-zcrop1.tif]

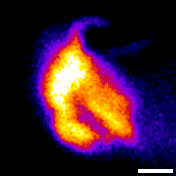

Supplement: Supplementary file 11 — Source data Fig. 5 [file 44318_2025_516_MOESM11_ESM.zip › Figure 5/Panel G/Control/082424_Prep3A_phalloidinA488-0.03%DMSO_Axon1_Section-002_ch00-zcrop2.tif]

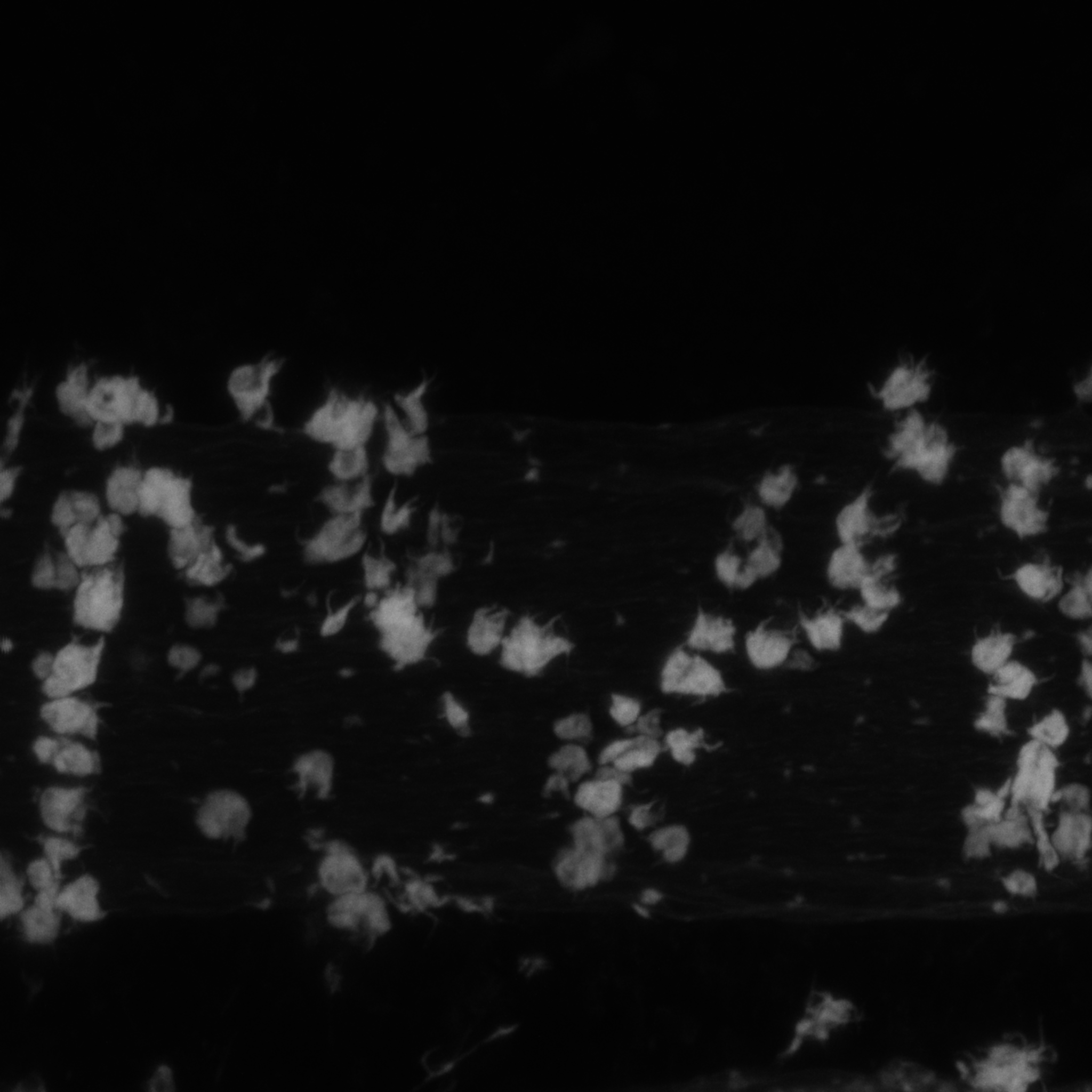

Supplement: Supplementary file 11 — Source data Fig. 5 [file 44318_2025_516_MOESM11_ESM.zip › Figure 5/Panel G/Control/MAX_082424_Prep3A_phalloidinA488-0.03%DMSO_Axon1_Section-002_ch00-z1-64.tif]

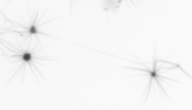

Supplement: Supplementary file 12 — Source data Fig. 6 [file 44318_2025_516_MOESM12_ESM.zip › Figure 6/Panel B/Network_Actin (MaxZProj)_Cutout.tif]

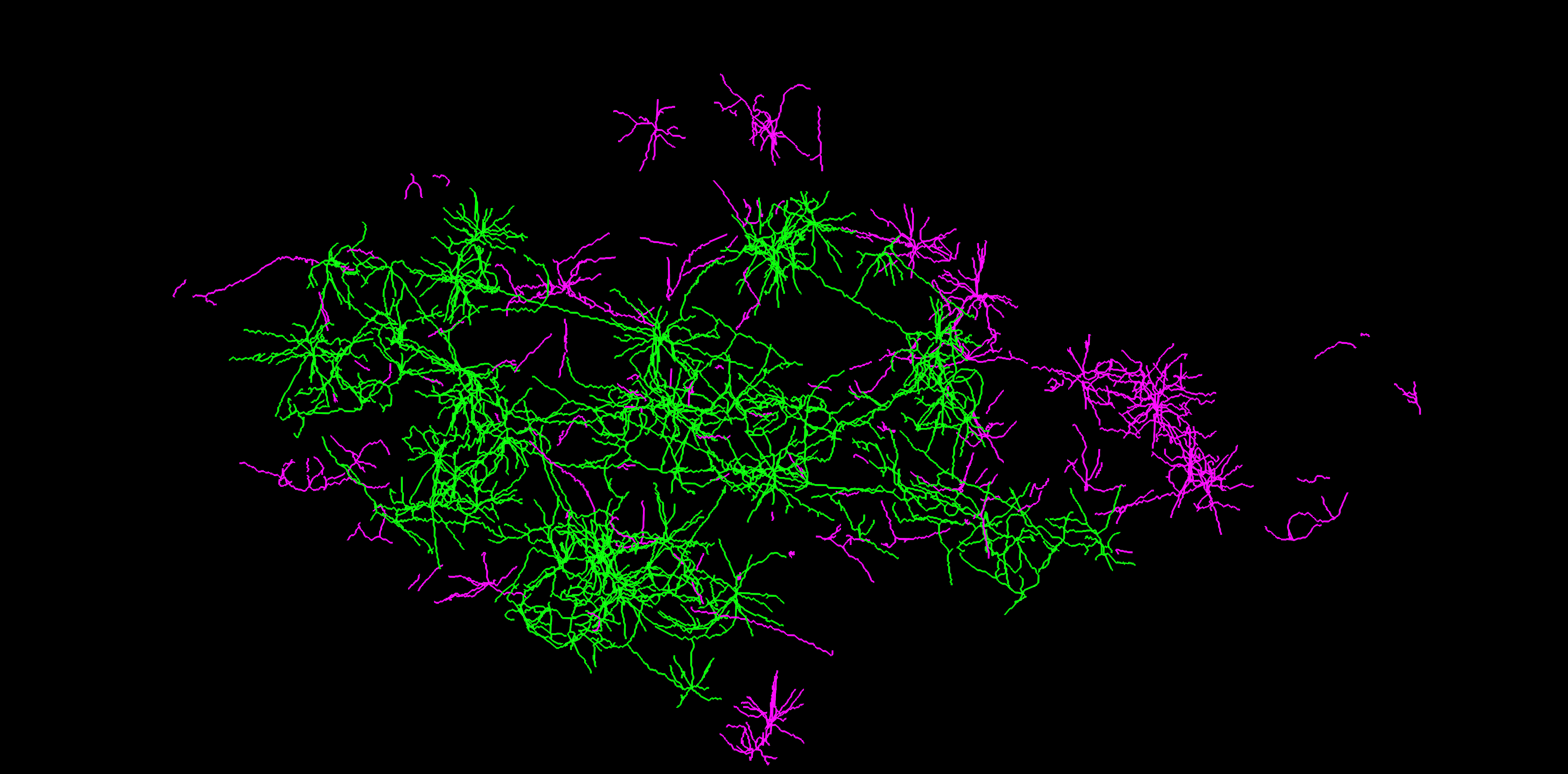

Supplement: Supplementary file 12 — Source data Fig. 6 [file 44318_2025_516_MOESM12_ESM.zip › Figure 6/Panel C/Interconnected Condensates Network.png]

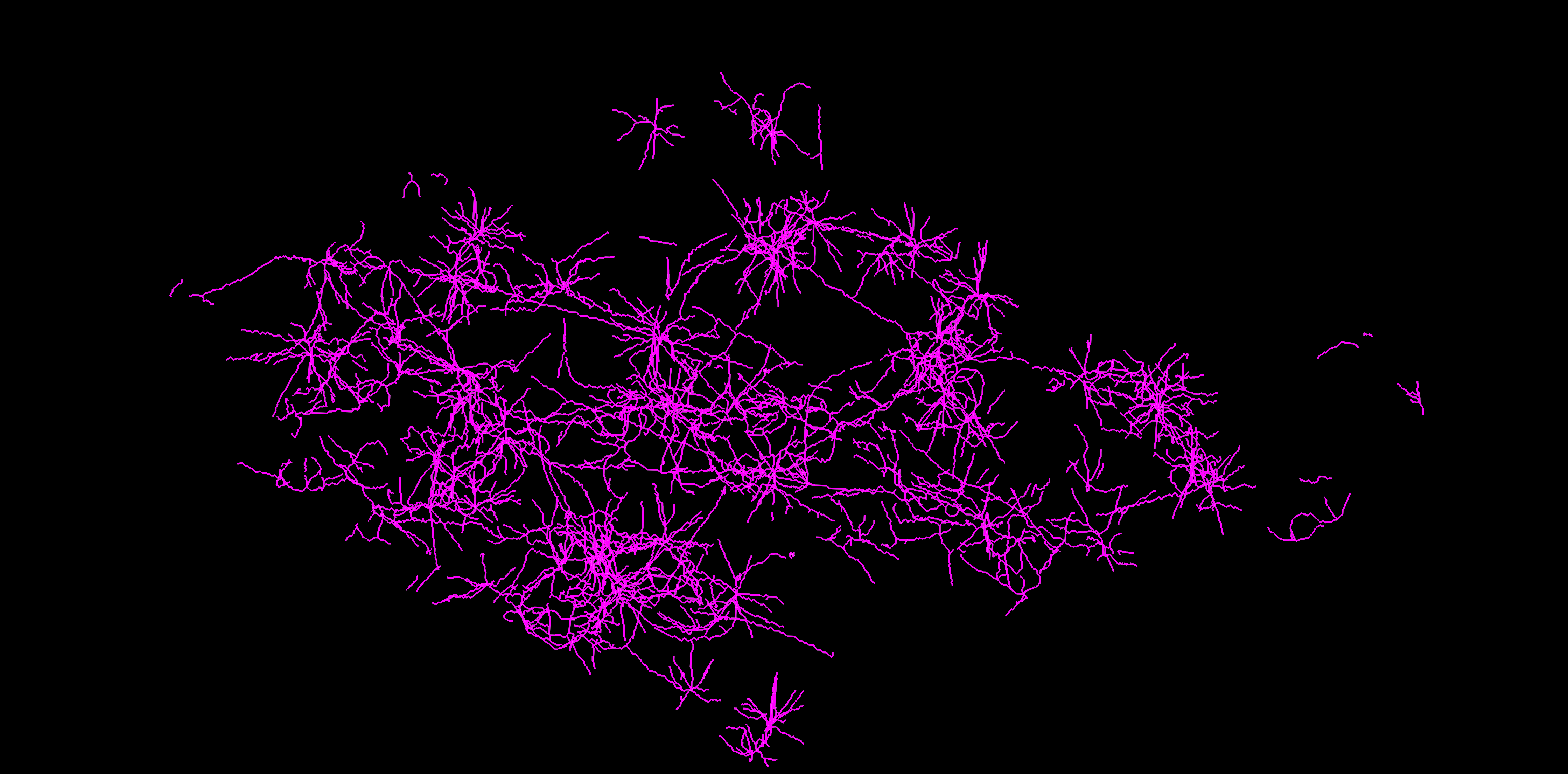

Supplement: Supplementary file 12 — Source data Fig. 6 [file 44318_2025_516_MOESM12_ESM.zip › Figure 6/Panel C/Skeletonized Actin Fibrils.png]

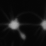

Supplement: Supplementary file 12 — Source data Fig. 6 [file 44318_2025_516_MOESM12_ESM.zip › Figure 6/Panel F/ZMAX_Crop_Actin.tiff]

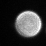

Supplement: Supplementary file 12 — Source data Fig. 6 [file 44318_2025_516_MOESM12_ESM.zip › Figure 6/Panel F/ZMAX_Crop_GUV.tiff]

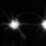

Supplement: Supplementary file 12 — Source data Fig. 6 [file 44318_2025_516_MOESM12_ESM.zip › Figure 6/Panel F/ZMAX_Crop_Synapsin1.tiff]

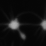

Supplement: Supplementary file 12 — Source data Fig. 6 [file 44318_2025_516_MOESM12_ESM.zip › Figure 6/Panel F/ZMAX_Crop_Composite.tif]

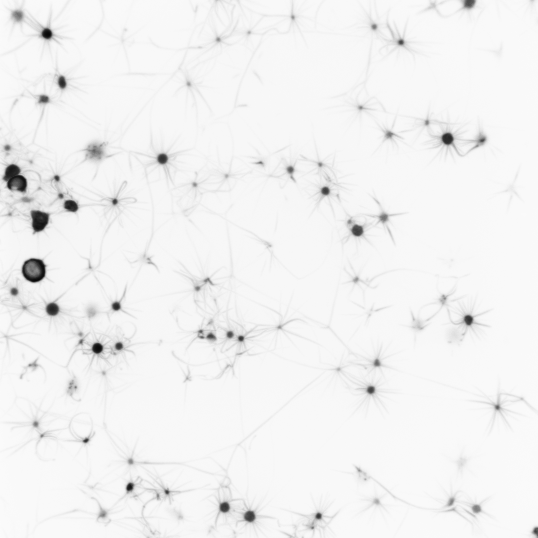

Supplement: Supplementary file 12 — Source data Fig. 6 [file 44318_2025_516_MOESM12_ESM.zip › Figure 6/Panel A/Network_Actin (MaxZProj).tiff]

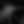

Supplement: Supplementary file 12 — Source data Fig. 6 [file 44318_2025_516_MOESM12_ESM.zip › Figure 6/Panel H/Before Hxd_Synapsin1_Crop.tif]

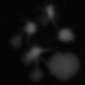

Supplement: Supplementary file 12 — Source data Fig. 6 [file 44318_2025_516_MOESM12_ESM.zip › Figure 6/Panel H/Before Hxd_Composite.tif]

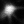

Supplement: Supplementary file 12 — Source data Fig. 6 [file 44318_2025_516_MOESM12_ESM.zip › Figure 6/Panel H/Before Hxd_Actin_Crop.tif]

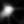

Supplement: Supplementary file 12 — Source data Fig. 6 [file 44318_2025_516_MOESM12_ESM.zip › Figure 6/Panel H/After Hxd_Synapsin1_Crop.tif]

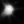

Supplement: Supplementary file 12 — Source data Fig. 6 [file 44318_2025_516_MOESM12_ESM.zip › Figure 6/Panel H/After Hxd_Actin_Crop.tif]

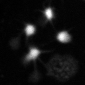

Supplement: Supplementary file 12 — Source data Fig. 6 [file 44318_2025_516_MOESM12_ESM.zip › Figure 6/Panel H/After Hxd_Composite.tif]

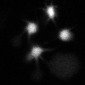

Supplement: Supplementary file 12 — Source data Fig. 6 [file 44318_2025_516_MOESM12_ESM.zip › Figure 6/Panel H/Before Hxd_Actin.tif]

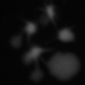

Supplement: Supplementary file 12 — Source data Fig. 6 [file 44318_2025_516_MOESM12_ESM.zip › Figure 6/Panel H/Before Hxd_Synapsin1.tif]

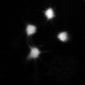

Supplement: Supplementary file 12 — Source data Fig. 6 [file 44318_2025_516_MOESM12_ESM.zip › Figure 6/Panel H/After Hxd_Actin.tif]

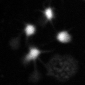

Supplement: Supplementary file 12 — Source data Fig. 6 [file 44318_2025_516_MOESM12_ESM.zip › Figure 6/Panel H/After Hxd_Synapsin.tif]
