# Supplementary figures and images for: Condensates of synaptic vesicles and synapsin-1 mediate actin sequestering and polymerization (part 2 of 3)
Source: EMBO J. 2025 Aug 14;44(18):5112–48. doi: 10.1038/s44318-025-00516-y (PMC12436662; doi:10.1038/s44318-025-00516-y)

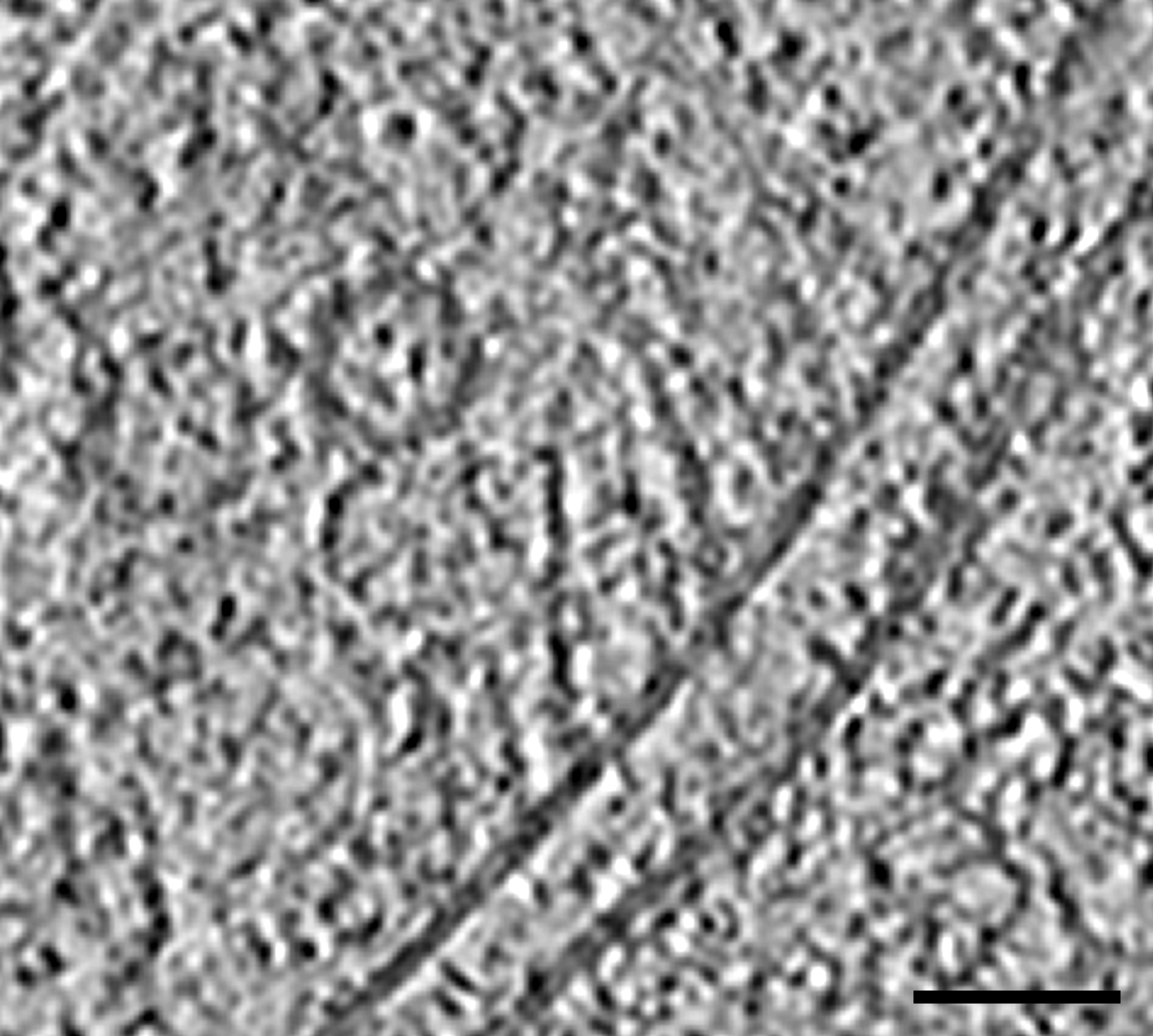

Supplement: Supplementary file 13 — Source data Fig. 7 [file 44318_2025_516_MOESM13_ESM.zip › Figure 7/Panel E/Figure7E.tif]

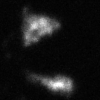

Supplement: Supplementary file 13 — Source data Fig. 7 [file 44318_2025_516_MOESM13_ESM.zip › Figure 7/Panel F/Cutout_VGLUT1.tif]

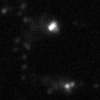

Supplement: Supplementary file 13 — Source data Fig. 7 [file 44318_2025_516_MOESM13_ESM.zip › Figure 7/Panel F/Cutout_PSD95.tif]

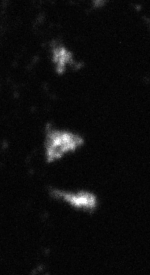

Supplement: Supplementary file 13 — Source data Fig. 7 [file 44318_2025_516_MOESM13_ESM.zip › Figure 7/Panel F/Neuronal segment_Composite.tif]

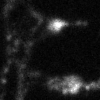

Supplement: Supplementary file 13 — Source data Fig. 7 [file 44318_2025_516_MOESM13_ESM.zip › Figure 7/Panel F/Cutout_Actin, increased contrast.tif]

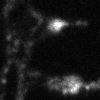

Supplement: Supplementary file 13 — Source data Fig. 7 [file 44318_2025_516_MOESM13_ESM.zip › Figure 7/Panel F/Cutout_Actin.tif]

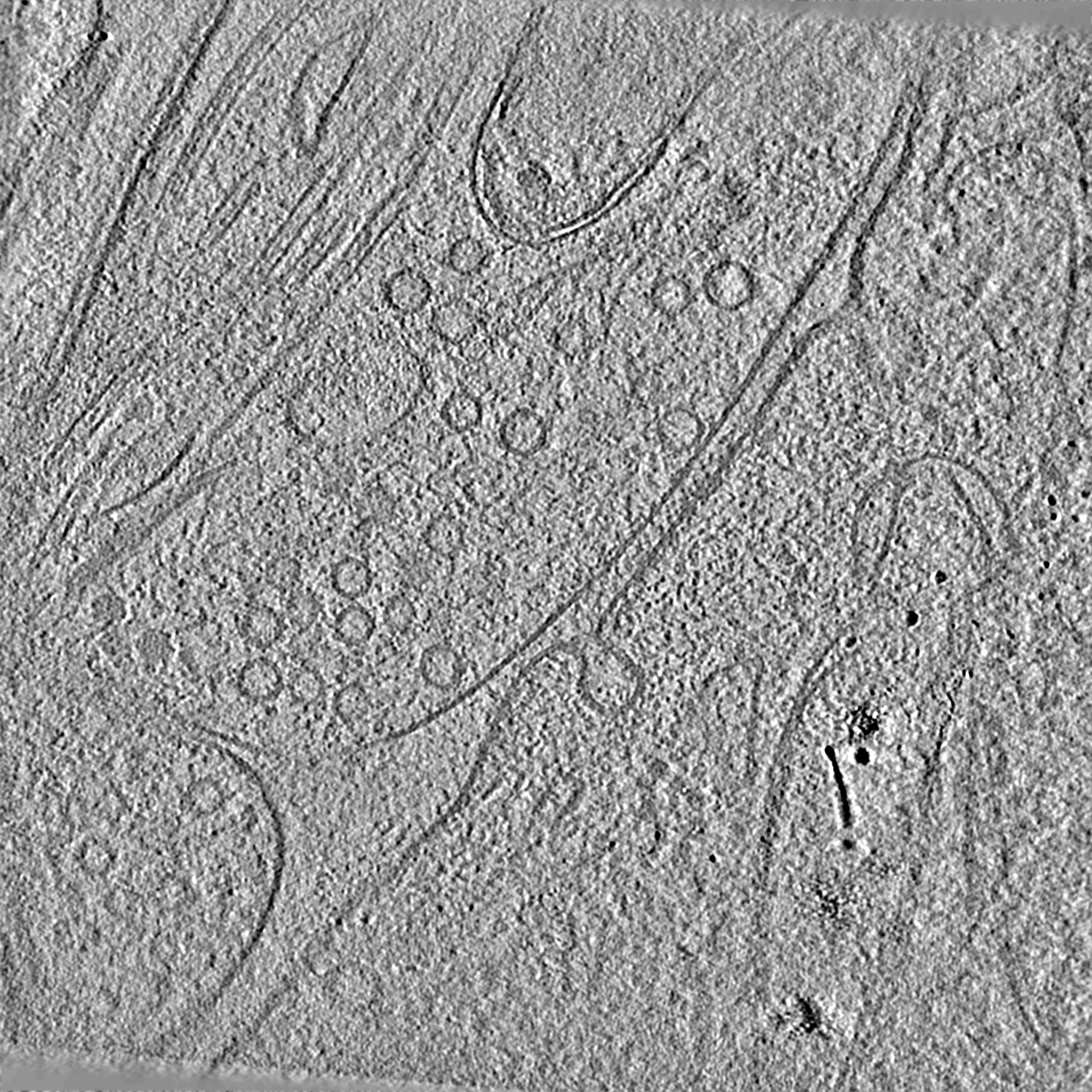

Supplement: Supplementary file 13 — Source data Fig. 7 [file 44318_2025_516_MOESM13_ESM.zip › Figure 7/Panel A/Figure7A.tif]

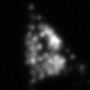

Supplement: Supplementary file 13 — Source data Fig. 7 [file 44318_2025_516_MOESM13_ESM.zip › Figure 7/Panel G/Cutout_VGLUT1.tif]

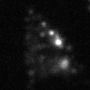

Supplement: Supplementary file 13 — Source data Fig. 7 [file 44318_2025_516_MOESM13_ESM.zip › Figure 7/Panel G/Cutout_PSD95.tif]

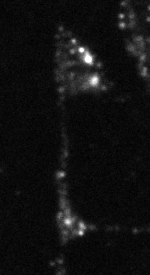

Supplement: Supplementary file 13 — Source data Fig. 7 [file 44318_2025_516_MOESM13_ESM.zip › Figure 7/Panel G/Neuronal segment_Composite.tif]

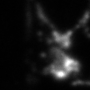

Supplement: Supplementary file 13 — Source data Fig. 7 [file 44318_2025_516_MOESM13_ESM.zip › Figure 7/Panel G/Cutout_Actin, increased contrast.tif]

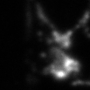

Supplement: Supplementary file 13 — Source data Fig. 7 [file 44318_2025_516_MOESM13_ESM.zip › Figure 7/Panel G/Cutout_Actin.tif]

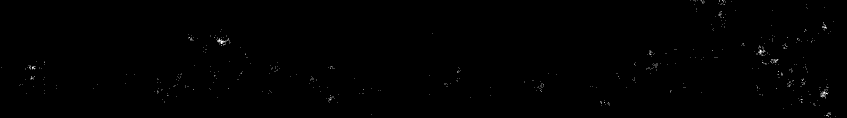

Supplement: Supplementary file 14 — Source data Fig. 8 [file 44318_2025_516_MOESM14_ESM.zip › Figure 8/Panel B/SYP.tif]

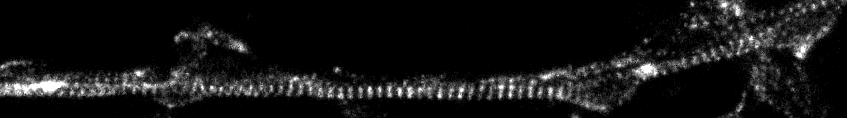

Supplement: Supplementary file 14 — Source data Fig. 8 [file 44318_2025_516_MOESM14_ESM.zip › Figure 8/Panel B/Actin.tif]

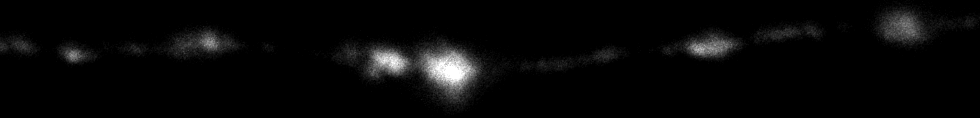

Supplement: Supplementary file 14 — Source data Fig. 8 [file 44318_2025_516_MOESM14_ESM.zip › Figure 8/Panel E/EGFP-Synapsin1.tif]

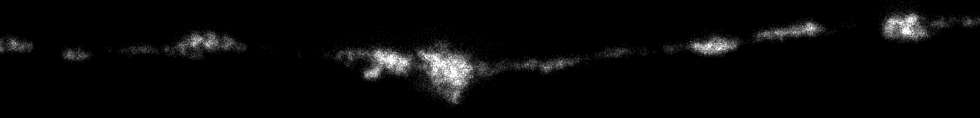

Supplement: Supplementary file 14 — Source data Fig. 8 [file 44318_2025_516_MOESM14_ESM.zip › Figure 8/Panel E/SYP.tif]

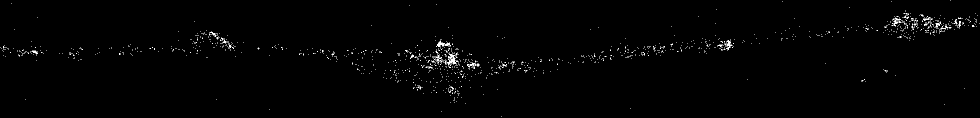

Supplement: Supplementary file 14 — Source data Fig. 8 [file 44318_2025_516_MOESM14_ESM.zip › Figure 8/Panel E/Actin.tif]

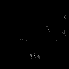

Supplement: Supplementary file 14 — Source data Fig. 8 [file 44318_2025_516_MOESM14_ESM.zip › Figure 8/Panel D/SYP.tif]

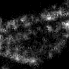

Supplement: Supplementary file 14 — Source data Fig. 8 [file 44318_2025_516_MOESM14_ESM.zip › Figure 8/Panel D/Actin.tif]

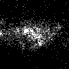

Supplement: Supplementary file 14 — Source data Fig. 8 [file 44318_2025_516_MOESM14_ESM.zip › Figure 8/Panel C/SYP.tif]

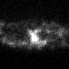

Supplement: Supplementary file 14 — Source data Fig. 8 [file 44318_2025_516_MOESM14_ESM.zip › Figure 8/Panel C/Actin.tif]

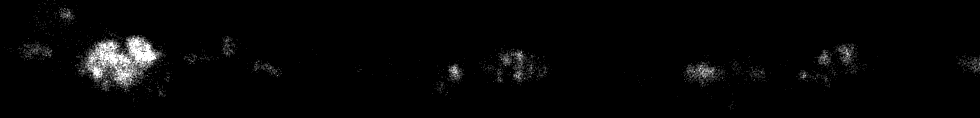

Supplement: Supplementary file 14 — Source data Fig. 8 [file 44318_2025_516_MOESM14_ESM.zip › Figure 8/Panel F/SYP.tif]

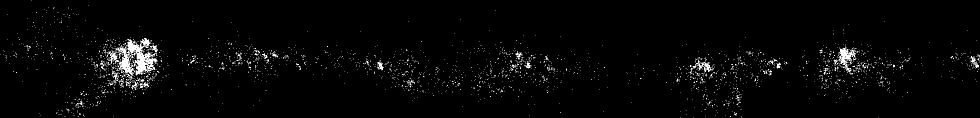

Supplement: Supplementary file 14 — Source data Fig. 8 [file 44318_2025_516_MOESM14_ESM.zip › Figure 8/Panel F/Actin.tif]

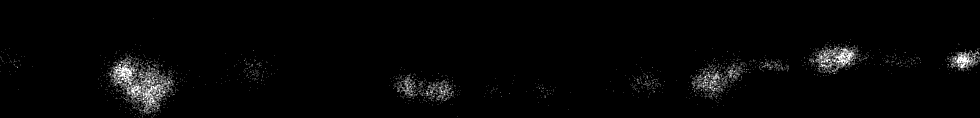

Supplement: Supplementary file 14 — Source data Fig. 8 [file 44318_2025_516_MOESM14_ESM.zip › Figure 8/Panel F/EGFP-IDR.tif]

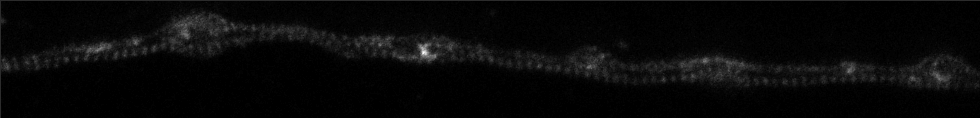

Supplement: Supplementary file 14 — Source data Fig. 8 [file 44318_2025_516_MOESM14_ESM.zip › Figure 8/Panel A/Composite.tif]

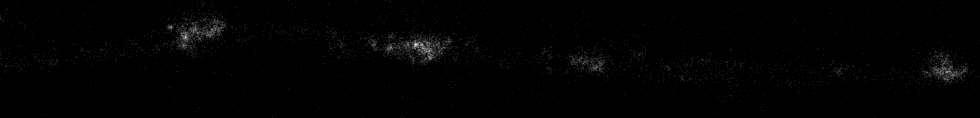

Supplement: Supplementary file 14 — Source data Fig. 8 [file 44318_2025_516_MOESM14_ESM.zip › Figure 8/Panel A/SYP.tif]

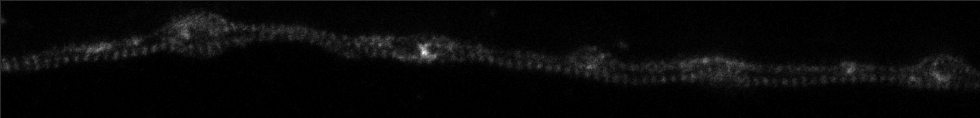

Supplement: Supplementary file 14 — Source data Fig. 8 [file 44318_2025_516_MOESM14_ESM.zip › Figure 8/Panel A/Actin.tif]

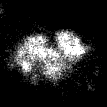

Supplement: Supplementary file 14 — Source data Fig. 8 [file 44318_2025_516_MOESM14_ESM.zip › Figure 8/Panel H/SYP.tif]

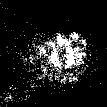

Supplement: Supplementary file 14 — Source data Fig. 8 [file 44318_2025_516_MOESM14_ESM.zip › Figure 8/Panel H/Actin.tif]

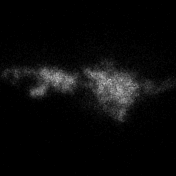

Supplement: Supplementary file 14 — Source data Fig. 8 [file 44318_2025_516_MOESM14_ESM.zip › Figure 8/Panel G/SYP.tif]

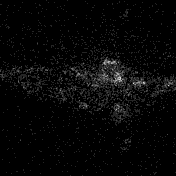

Supplement: Supplementary file 14 — Source data Fig. 8 [file 44318_2025_516_MOESM14_ESM.zip › Figure 8/Panel G/Actin.tif]

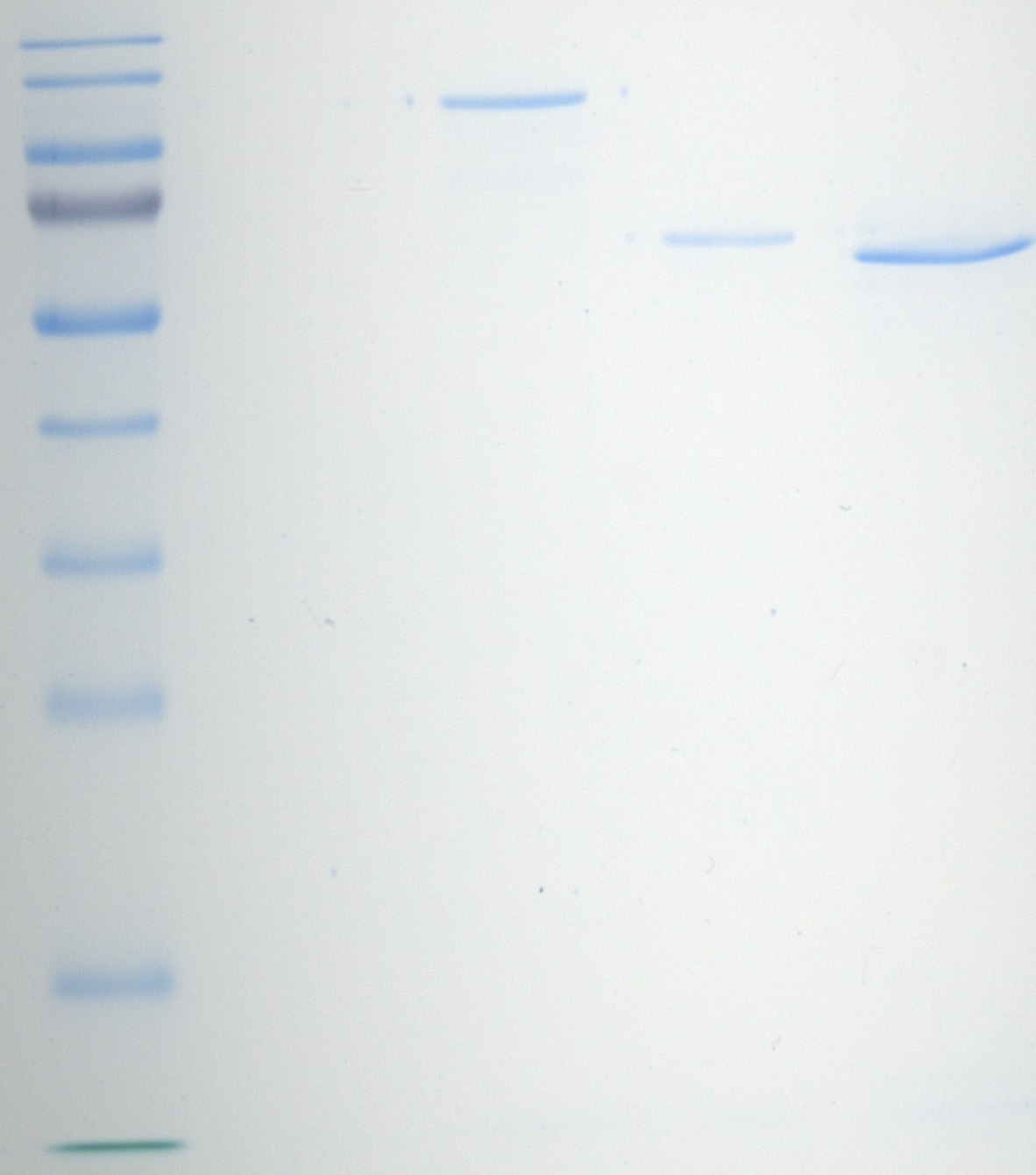

Supplement: Supplementary file 15 — Figure EV1 Source Data [file 44318_2025_516_MOESM15_ESM.zip › EV 1/Syn1-FL_IDR and Dom C (RGB)-1.tif]

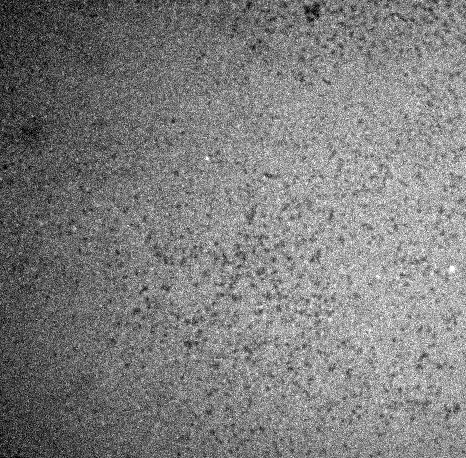

Supplement: Supplementary file 16 — Figure EV2 Source Data [file 44318_2025_516_MOESM16_ESM.zip › EV 2/Panel B/2 min/Syn1_MAX_Rep2_4┬╡M_actin-atp_syn1_002-1.tif]

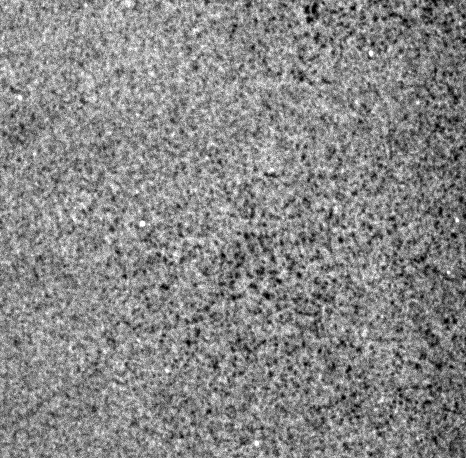

Supplement: Supplementary file 16 — Figure EV2 Source Data [file 44318_2025_516_MOESM16_ESM.zip › EV 2/Panel B/2 min/Actin_MAX_Rep2_4┬╡M_actin-atp_syn1_002-1.tif]

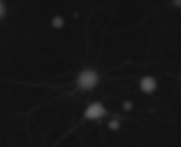

Supplement: Supplementary file 16 — Figure EV2 Source Data [file 44318_2025_516_MOESM16_ESM.zip › EV 2/Panel B/Magnified region/Syn1-Magenta LUT.tif]

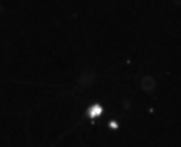

Supplement: Supplementary file 16 — Figure EV2 Source Data [file 44318_2025_516_MOESM16_ESM.zip › EV 2/Panel B/Magnified region/Actin-Green LUT.tif]

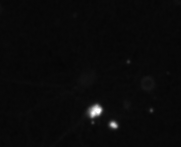

Supplement: Supplementary file 16 — Figure EV2 Source Data [file 44318_2025_516_MOESM16_ESM.zip › EV 2/Panel B/Magnified region/Actin-FIRE LUT.tif]

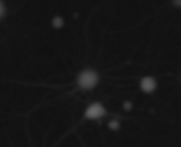

Supplement: Supplementary file 16 — Figure EV2 Source Data [file 44318_2025_516_MOESM16_ESM.zip › EV 2/Panel B/Magnified region/Syn1-FIRE LUT.tif]

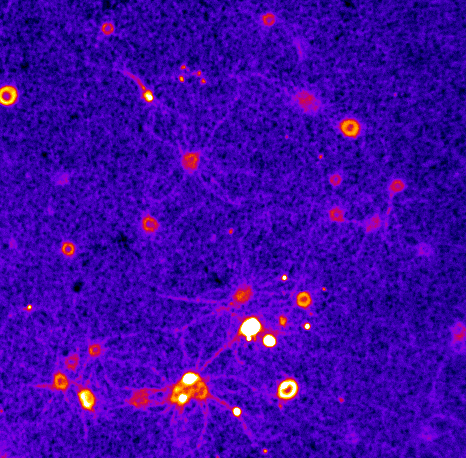

Supplement: Supplementary file 16 — Figure EV2 Source Data [file 44318_2025_516_MOESM16_ESM.zip › EV 2/Panel B/30 min/Actin_Rep3_4┬╡M_actin-atp_syn1_+3p-peg_before-lat--a_021-FIRE.tif]

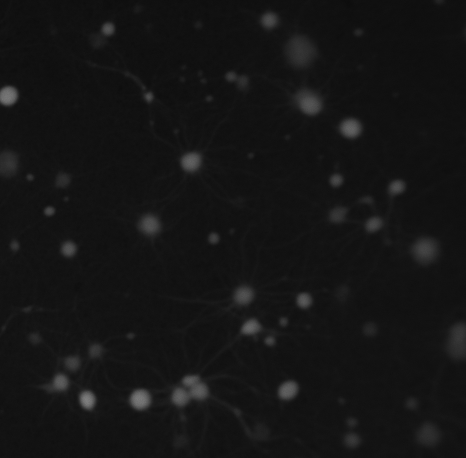

Supplement: Supplementary file 16 — Figure EV2 Source Data [file 44318_2025_516_MOESM16_ESM.zip › EV 2/Panel B/30 min/Syn1_Rep3_4┬╡M_actin-atp_syn1_+3p-peg_before-lat--a_021-fire.tif]

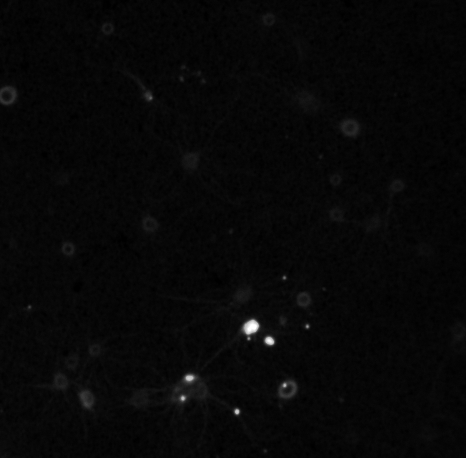

Supplement: Supplementary file 16 — Figure EV2 Source Data [file 44318_2025_516_MOESM16_ESM.zip › EV 2/Panel B/30 min/Actin_Rep3_4┬╡M_actin-atp_syn1_+3p-peg_before-lat--a_021.tif]

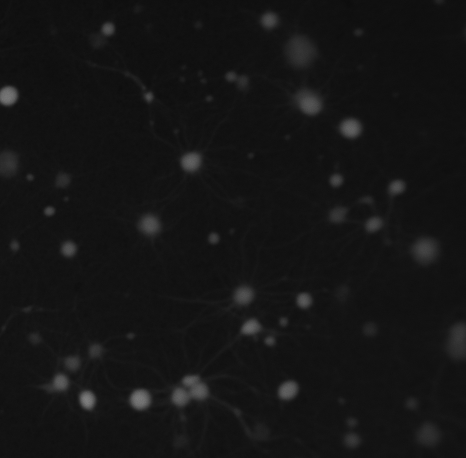

Supplement: Supplementary file 16 — Figure EV2 Source Data [file 44318_2025_516_MOESM16_ESM.zip › EV 2/Panel B/30 min/Syn1_Rep3_4┬╡M_actin-atp_syn1_+3p-peg_before-lat--a_021.tif]

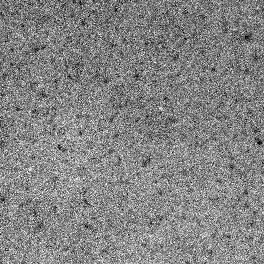

Supplement: Supplementary file 16 — Figure EV2 Source Data [file 44318_2025_516_MOESM16_ESM.zip › EV 2/Panel A/2 min/Syn1_MAX_4┬╡M_Syn1WT_rep3_4┬╡M_actin_002-1.tif]

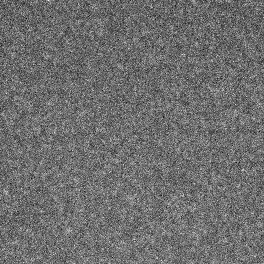

Supplement: Supplementary file 16 — Figure EV2 Source Data [file 44318_2025_516_MOESM16_ESM.zip › EV 2/Panel A/2 min/Actin_MAX_4┬╡M_Syn1WT_rep3_4┬╡M_actin_002-1.tif]

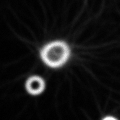

Supplement: Supplementary file 16 — Figure EV2 Source Data [file 44318_2025_516_MOESM16_ESM.zip › EV 2/Panel A/Magnified Region/Actin_Green_300 to 6000_Crop.tif]

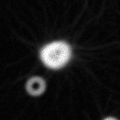

Supplement: Supplementary file 16 — Figure EV2 Source Data [file 44318_2025_516_MOESM16_ESM.zip › EV 2/Panel A/Magnified Region/Syn1_Magenta_300 to 4000_Crop.tif]

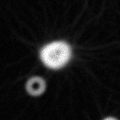

Supplement: Supplementary file 16 — Figure EV2 Source Data [file 44318_2025_516_MOESM16_ESM.zip › EV 2/Panel A/Magnified Region/Syn1_Fire_300 to 4000_Crop.tif]

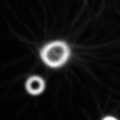

Supplement: Supplementary file 16 — Figure EV2 Source Data [file 44318_2025_516_MOESM16_ESM.zip › EV 2/Panel A/Magnified Region/Actin_Fire_300 to 6000_Crop.tif]

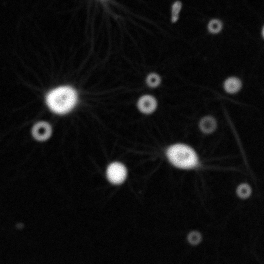

Supplement: Supplementary file 16 — Figure EV2 Source Data [file 44318_2025_516_MOESM16_ESM.zip › EV 2/Panel A/30 min/Syn1_4┬╡M_Syn1WT_rep3_4┬╡M_actin-atp_+3p-peg_007-1-1- fire LUT_01.tif]

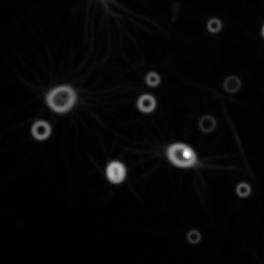

Supplement: Supplementary file 16 — Figure EV2 Source Data [file 44318_2025_516_MOESM16_ESM.zip › EV 2/Panel A/30 min/Actin_4┬╡M_Syn1WT_rep3_4┬╡M_actin-atp_+3p-peg_007-1-1.tif]

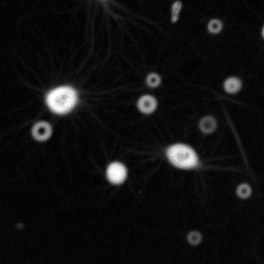

Supplement: Supplementary file 16 — Figure EV2 Source Data [file 44318_2025_516_MOESM16_ESM.zip › EV 2/Panel A/30 min/Syn1_4┬╡M_Syn1WT_rep3_4┬╡M_actin-atp_+3p-peg_007-1-1.tif]

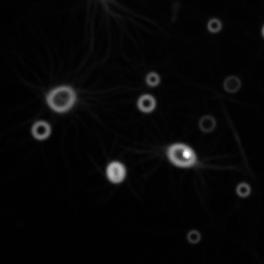

Supplement: Supplementary file 16 — Figure EV2 Source Data [file 44318_2025_516_MOESM16_ESM.zip › EV 2/Panel A/30 min/Actin_4┬╡M_Syn1WT_rep3_4┬╡M_actin-atp_+3p-peg_007-1-1- fire LUT_01.tif]

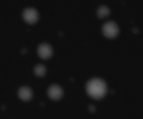

Supplement: Supplementary file 17 — Figure EV3 Source Data [file 44318_2025_516_MOESM17_ESM.zip › EV 3/Panel B/Syn1 at 35 min.tif]

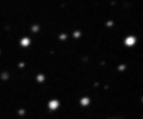

Supplement: Supplementary file 17 — Figure EV3 Source Data [file 44318_2025_516_MOESM17_ESM.zip › EV 3/Panel B/Syn1 at 0 min.tif]

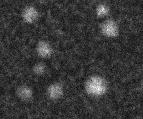

Supplement: Supplementary file 17 — Figure EV3 Source Data [file 44318_2025_516_MOESM17_ESM.zip › EV 3/Panel B/Actin at 35 min.tif]

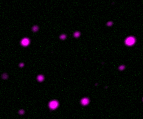

Supplement: Supplementary file 17 — Figure EV3 Source Data [file 44318_2025_516_MOESM17_ESM.zip › EV 3/Panel B/Merge at 0 min.tif]

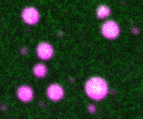

Supplement: Supplementary file 17 — Figure EV3 Source Data [file 44318_2025_516_MOESM17_ESM.zip › EV 3/Panel B/Merge at 35 min.tif]

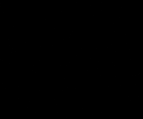

Supplement: Supplementary file 17 — Figure EV3 Source Data [file 44318_2025_516_MOESM17_ESM.zip › EV 3/Panel B/Actin at 0 min.tif]

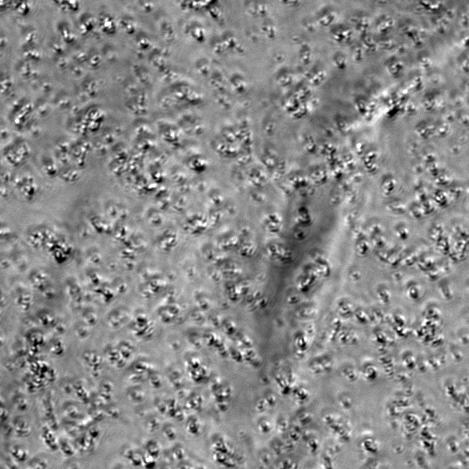

Supplement: Supplementary file 17 — Figure EV3 Source Data [file 44318_2025_516_MOESM17_ESM.zip › EV 3/Panel D/Syn1_FL_brightfield.tif]

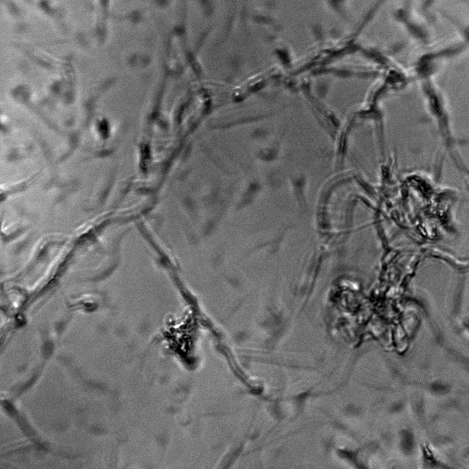

Supplement: Supplementary file 17 — Figure EV3 Source Data [file 44318_2025_516_MOESM17_ESM.zip › EV 3/Panel D/Syn1_FL + Actin_brightfield.tif]

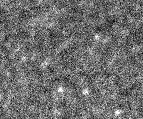

Supplement: Supplementary file 17 — Figure EV3 Source Data [file 44318_2025_516_MOESM17_ESM.zip › EV 3/Panel A/Actin at 35 min.tif]

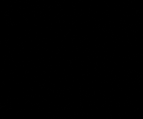

Supplement: Supplementary file 17 — Figure EV3 Source Data [file 44318_2025_516_MOESM17_ESM.zip › EV 3/Panel A/Merge at 0 min.tif]

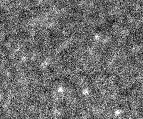

Supplement: Supplementary file 17 — Figure EV3 Source Data [file 44318_2025_516_MOESM17_ESM.zip › EV 3/Panel A/Merge at 35 min.tif]

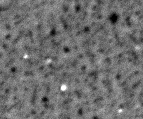

Supplement: Supplementary file 17 — Figure EV3 Source Data [file 44318_2025_516_MOESM17_ESM.zip › EV 3/Panel A/Syn1-DomC at 35 min.tif]

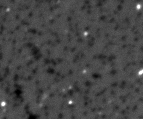

Supplement: Supplementary file 17 — Figure EV3 Source Data [file 44318_2025_516_MOESM17_ESM.zip › EV 3/Panel A/Syn1-DomC at 0 min.tif]

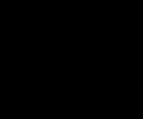

Supplement: Supplementary file 17 — Figure EV3 Source Data [file 44318_2025_516_MOESM17_ESM.zip › EV 3/Panel A/Actin at 0 min.tif]

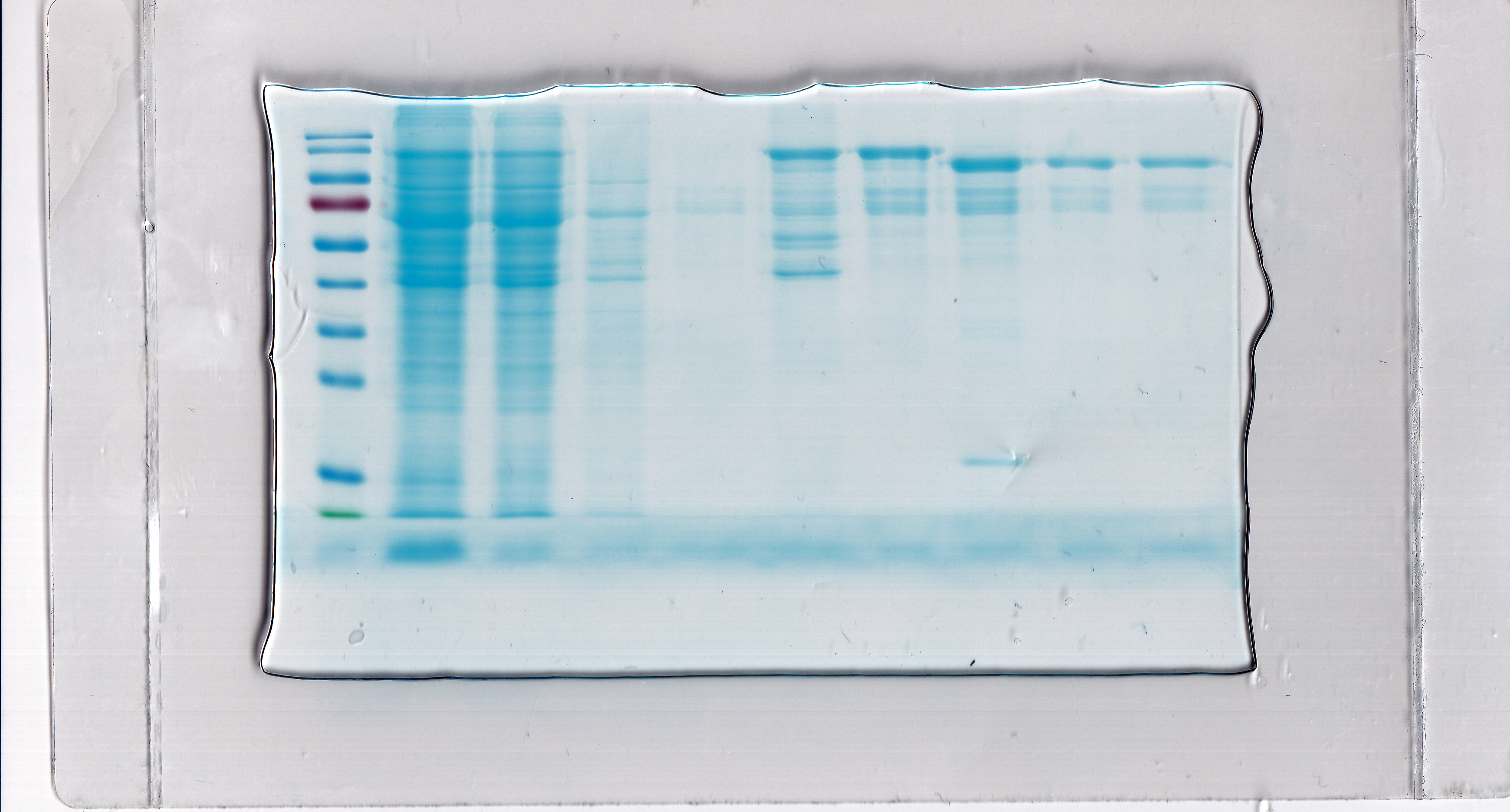

Supplement: Supplementary file 18 — Figure EV4 Source Data [file 44318_2025_516_MOESM18_ESM.zip › EV 4/Panel B/His_SUMO_EGFP_Syn1a_S568_605E_CaMKII_purification gel.tif]

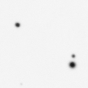

Supplement: Supplementary file 18 — Figure EV4 Source Data [file 44318_2025_516_MOESM18_ESM.zip › EV 4/Panel D/Syn1a_PKA+CaMKII_10min_zoom in.tif]

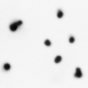

Supplement: Supplementary file 18 — Figure EV4 Source Data [file 44318_2025_516_MOESM18_ESM.zip › EV 4/Panel D/Syn1a_PKA_25min_zoom in.tif]

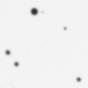

Supplement: Supplementary file 18 — Figure EV4 Source Data [file 44318_2025_516_MOESM18_ESM.zip › EV 4/Panel D/Syn1a_WT_10min_zoom in.tif]

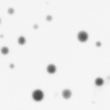

Supplement: Supplementary file 18 — Figure EV4 Source Data [file 44318_2025_516_MOESM18_ESM.zip › EV 4/Panel D/Syn1a_CamKII_25min_zoom in.tif]

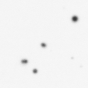

Supplement: Supplementary file 18 — Figure EV4 Source Data [file 44318_2025_516_MOESM18_ESM.zip › EV 4/Panel D/Syn1a_PKA+CamKII_25min_zoom in.tif]

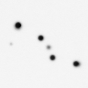

Supplement: Supplementary file 18 — Figure EV4 Source Data [file 44318_2025_516_MOESM18_ESM.zip › EV 4/Panel D/Syn1a_PKA_10min_zoom in.tif]

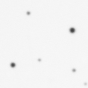

Supplement: Supplementary file 18 — Figure EV4 Source Data [file 44318_2025_516_MOESM18_ESM.zip › EV 4/Panel D/Syn1a_CamKII_10min_zoom in.tif]

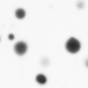

Supplement: Supplementary file 18 — Figure EV4 Source Data [file 44318_2025_516_MOESM18_ESM.zip › EV 4/Panel D/Syn1a_WT_25min_zoom in.tif]

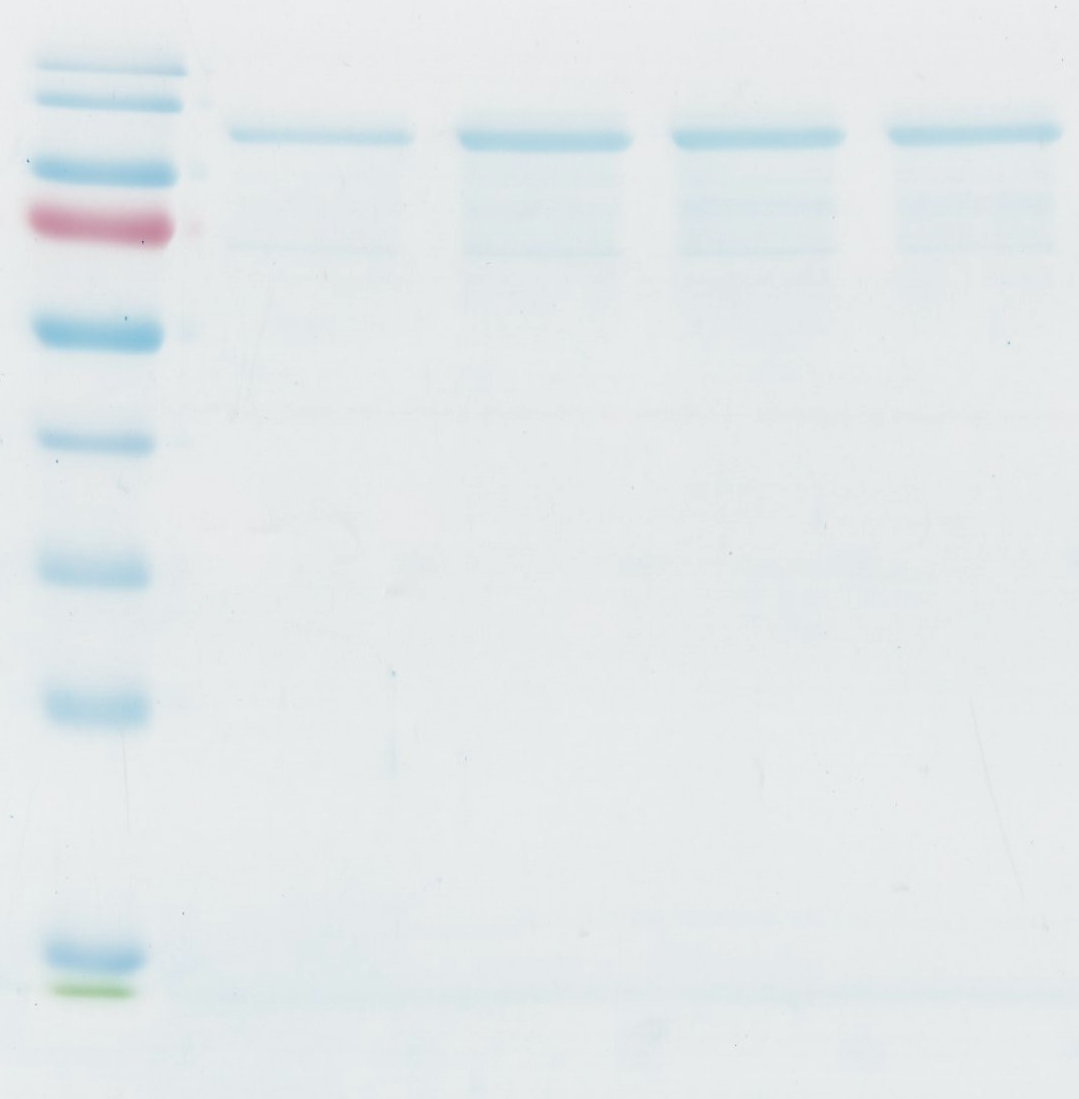

Supplement: Supplementary file 18 — Figure EV4 Source Data [file 44318_2025_516_MOESM18_ESM.zip › EV 4/Panel C/Syn1 WT and phosphomimetics_all proteins gel.tif]

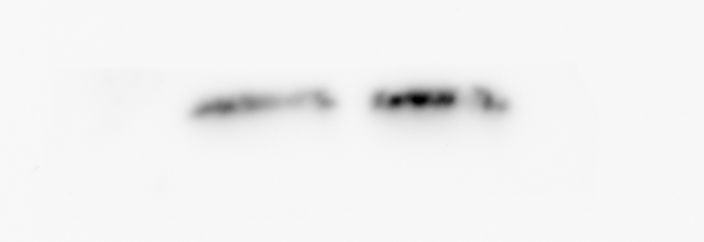

Supplement: Supplementary file 19 — Figure EV5 Source Data [file 44318_2025_516_MOESM19_ESM.zip › EV 5/Panel B/Rab5.tif]

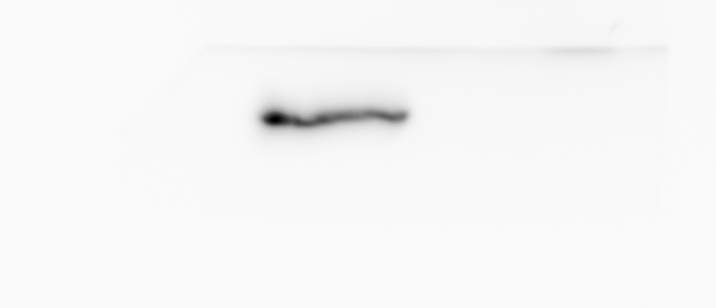

Supplement: Supplementary file 19 — Figure EV5 Source Data [file 44318_2025_516_MOESM19_ESM.zip › EV 5/Panel B/beta-actin.tif]

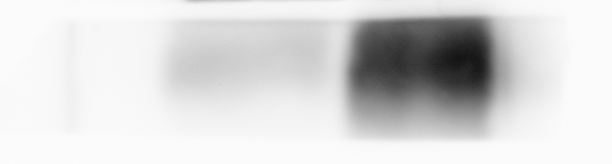

Supplement: Supplementary file 19 — Figure EV5 Source Data [file 44318_2025_516_MOESM19_ESM.zip › EV 5/Panel B/VGLUT1.tif]

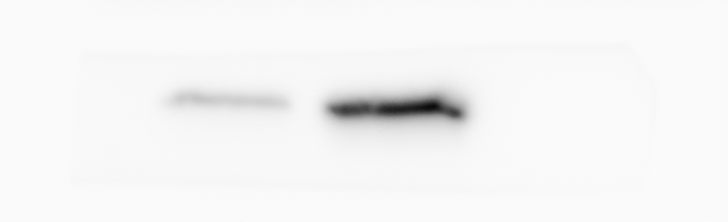

Supplement: Supplementary file 19 — Figure EV5 Source Data [file 44318_2025_516_MOESM19_ESM.zip › EV 5/Panel B/Rab3a.tif]

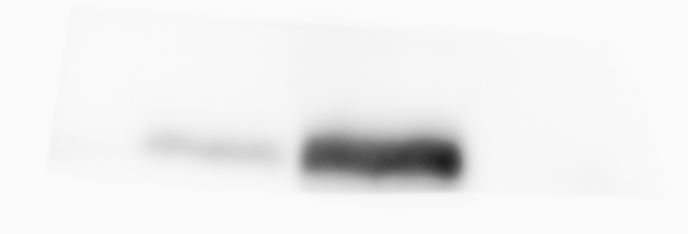

Supplement: Supplementary file 19 — Figure EV5 Source Data [file 44318_2025_516_MOESM19_ESM.zip › EV 5/Panel B/Synaptophysin 1.tif]

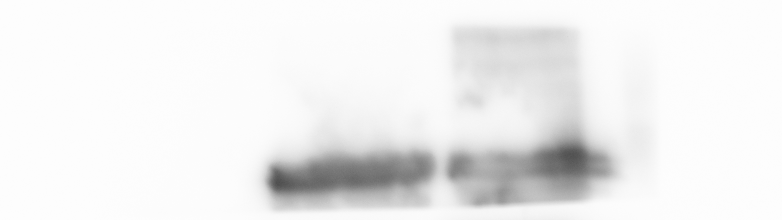

Supplement: Supplementary file 19 — Figure EV5 Source Data [file 44318_2025_516_MOESM19_ESM.zip › EV 5/Panel B/Synapsin 1.tif]

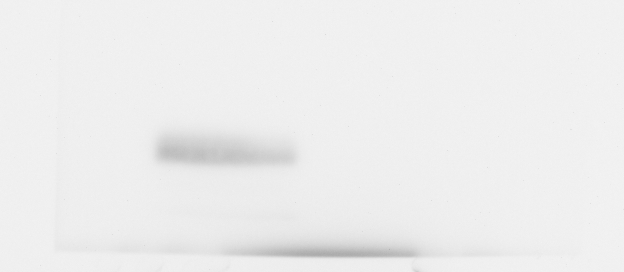

Supplement: Supplementary file 19 — Figure EV5 Source Data [file 44318_2025_516_MOESM19_ESM.zip › EV 5/Panel B/PSD95.tif]

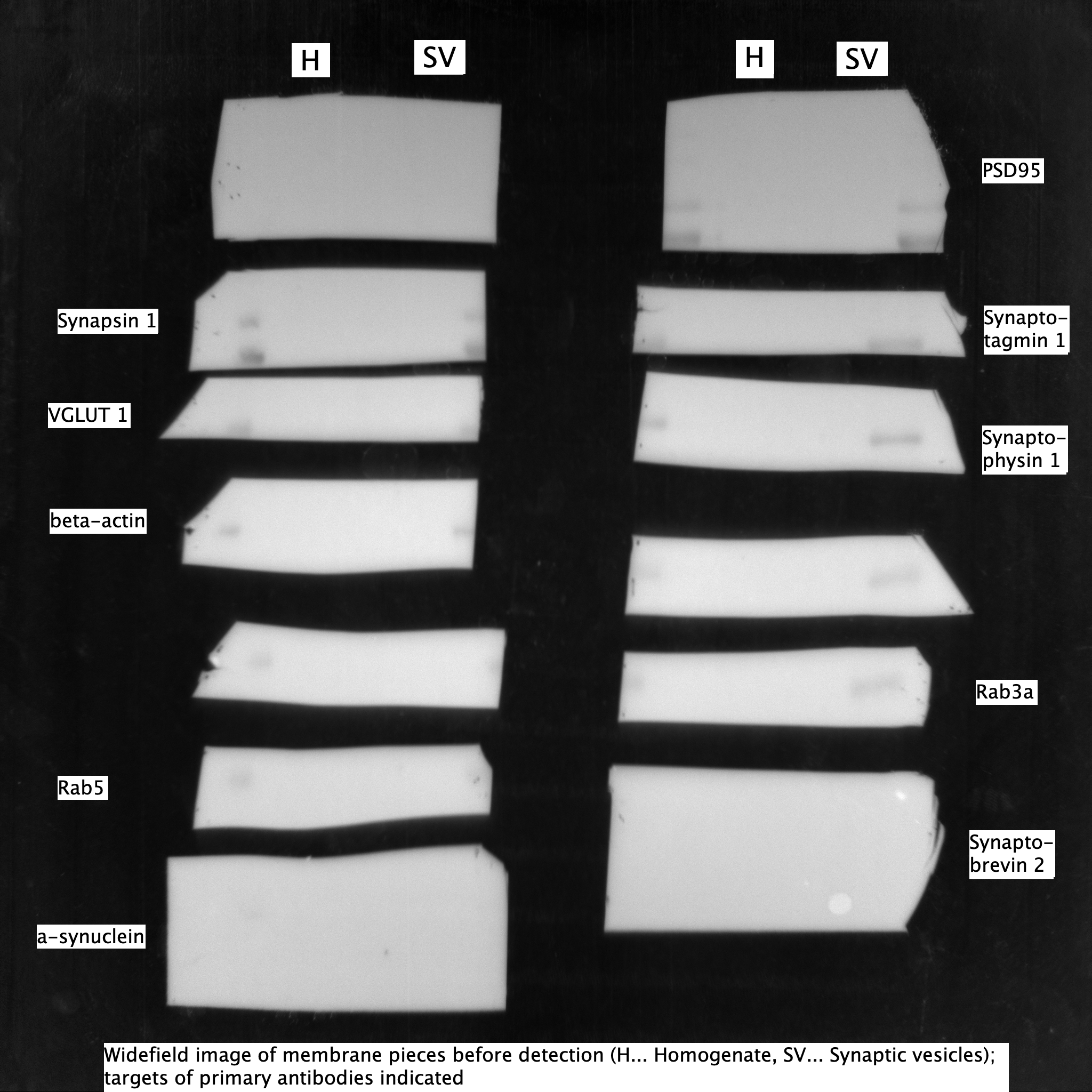

Supplement: Supplementary file 19 — Figure EV5 Source Data [file 44318_2025_516_MOESM19_ESM.zip › EV 5/Panel B/1_SV_Western_Blot_Overlay_Widefield.tif]

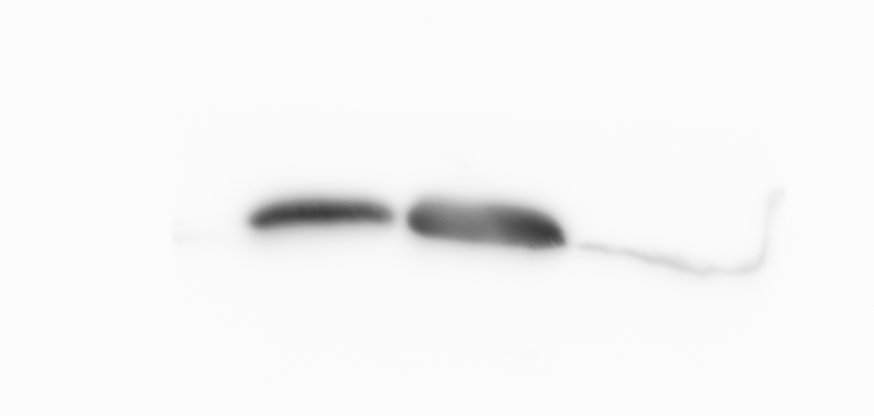

Supplement: Supplementary file 19 — Figure EV5 Source Data [file 44318_2025_516_MOESM19_ESM.zip › EV 5/Panel B/Synaptobrevin 2.tif]

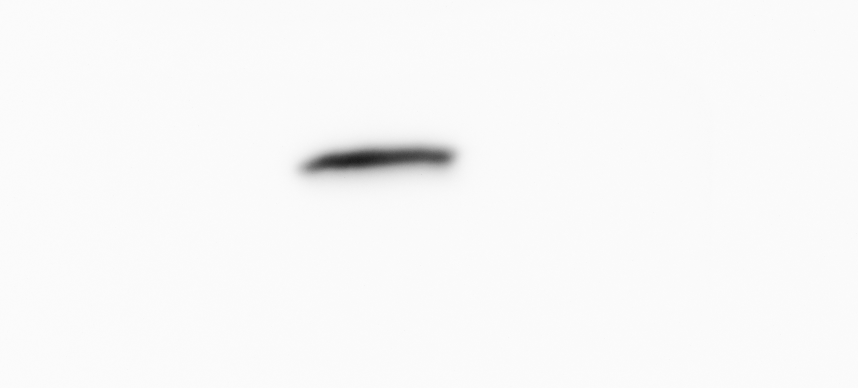

Supplement: Supplementary file 19 — Figure EV5 Source Data [file 44318_2025_516_MOESM19_ESM.zip › EV 5/Panel B/alpha-synuclein.tif]

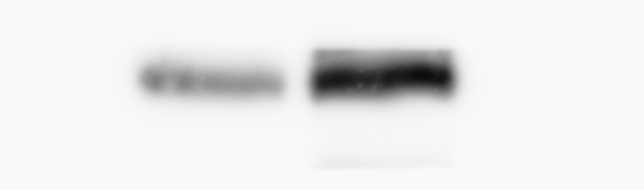

Supplement: Supplementary file 19 — Figure EV5 Source Data [file 44318_2025_516_MOESM19_ESM.zip › EV 5/Panel B/Synaptotagmin 1.tif]

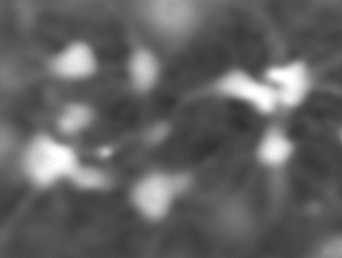

Supplement: Supplementary file 19 — Figure EV5 Source Data [file 44318_2025_516_MOESM19_ESM.zip › EV 5/Panel D/Syn1-IDR at 90 min.tif]

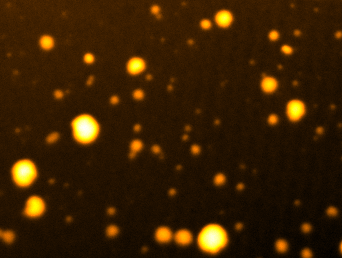

Supplement: Supplementary file 19 — Figure EV5 Source Data [file 44318_2025_516_MOESM19_ESM.zip › EV 5/Panel D/SVs at 0 min.tif]

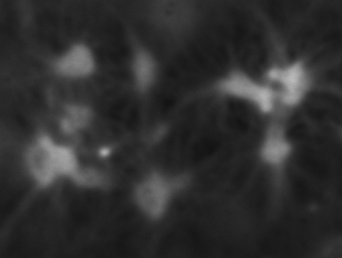

Supplement: Supplementary file 19 — Figure EV5 Source Data [file 44318_2025_516_MOESM19_ESM.zip › EV 5/Panel D/SVs at 90 min.tif]

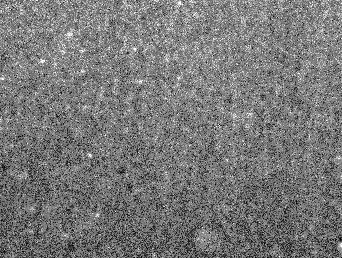

Supplement: Supplementary file 19 — Figure EV5 Source Data [file 44318_2025_516_MOESM19_ESM.zip › EV 5/Panel D/Merge at 0 min.tif]

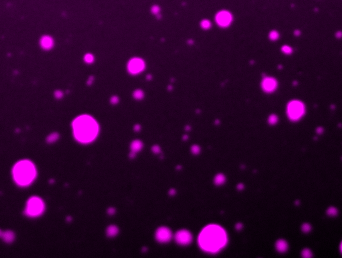

Supplement: Supplementary file 19 — Figure EV5 Source Data [file 44318_2025_516_MOESM19_ESM.zip › EV 5/Panel D/Syn1-IDR at 0 min.tif]

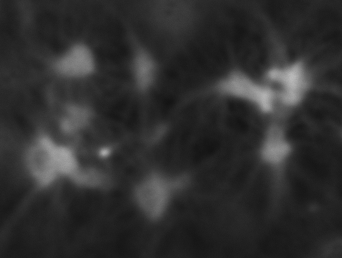

Supplement: Supplementary file 19 — Figure EV5 Source Data [file 44318_2025_516_MOESM19_ESM.zip › EV 5/Panel D/Merge at 90 min.tif]

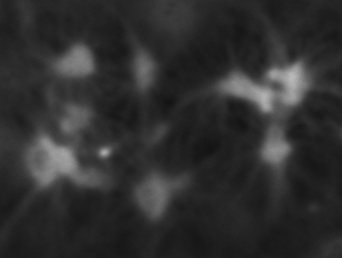

Supplement: Supplementary file 19 — Figure EV5 Source Data [file 44318_2025_516_MOESM19_ESM.zip › EV 5/Panel D/Actin at 90 min.tif]

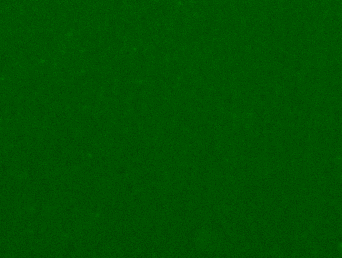

Supplement: Supplementary file 19 — Figure EV5 Source Data [file 44318_2025_516_MOESM19_ESM.zip › EV 5/Panel D/Actin at 0 min.tif]

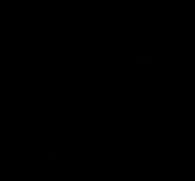

Supplement: Supplementary file 19 — Figure EV5 Source Data [file 44318_2025_516_MOESM19_ESM.zip › EV 5/Panel A/SVs at 0 min.tif]

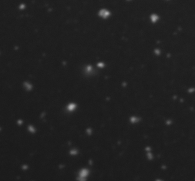

Supplement: Supplementary file 19 — Figure EV5 Source Data [file 44318_2025_516_MOESM19_ESM.zip › EV 5/Panel A/Actin at 45 min.tif]

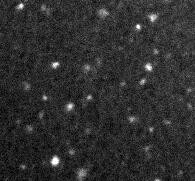

Supplement: Supplementary file 19 — Figure EV5 Source Data [file 44318_2025_516_MOESM19_ESM.zip › EV 5/Panel A/SVs at 45 min.tif]

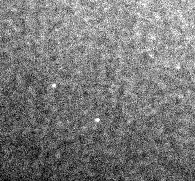

Supplement: Supplementary file 19 — Figure EV5 Source Data [file 44318_2025_516_MOESM19_ESM.zip › EV 5/Panel A/Actin at 0 min.tif]

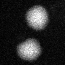

Supplement: Supplementary file 20 — Figure EV6 Source Data [file 44318_2025_516_MOESM20_ESM.zip › EV 6/Panel A/Cutout_BeforeHxd_Merge.tif]
