# Supplementary figures and images for: Condensates of synaptic vesicles and synapsin-1 mediate actin sequestering and polymerization (part 3 of 3)
Source: EMBO J. 2025 Aug 14;44(18):5112–48. doi: 10.1038/s44318-025-00516-y (PMC12436662; doi:10.1038/s44318-025-00516-y)

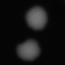

Supplement: Supplementary file 20 — Figure EV6 Source Data [file 44318_2025_516_MOESM20_ESM.zip › EV 6/Panel A/Cutout_BeforeHxd_Syn1_30000.tif]

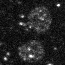

Supplement: Supplementary file 20 — Figure EV6 Source Data [file 44318_2025_516_MOESM20_ESM.zip › EV 6/Panel A/Cutout_AfterHxd_Actin_2100.tif]

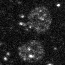

Supplement: Supplementary file 20 — Figure EV6 Source Data [file 44318_2025_516_MOESM20_ESM.zip › EV 6/Panel A/Cutout_AfterHxd_Merge.tif]

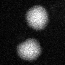

Supplement: Supplementary file 20 — Figure EV6 Source Data [file 44318_2025_516_MOESM20_ESM.zip › EV 6/Panel A/Cutout_BeforeHxd_Actin_2100.tif]

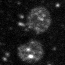

Supplement: Supplementary file 20 — Figure EV6 Source Data [file 44318_2025_516_MOESM20_ESM.zip › EV 6/Panel A/Cutout_AfterHxd_Syn1_30000.tif]

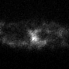

Supplement: Supplementary file 21 — Figure EV7 Source Data [file 44318_2025_516_MOESM21_ESM.zip › EV 7/Panel A/WT_Cutout.tif]

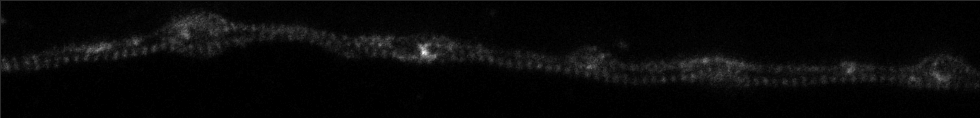

Supplement: Supplementary file 21 — Figure EV7 Source Data [file 44318_2025_516_MOESM21_ESM.zip › EV 7/Panel A/WT.tif]

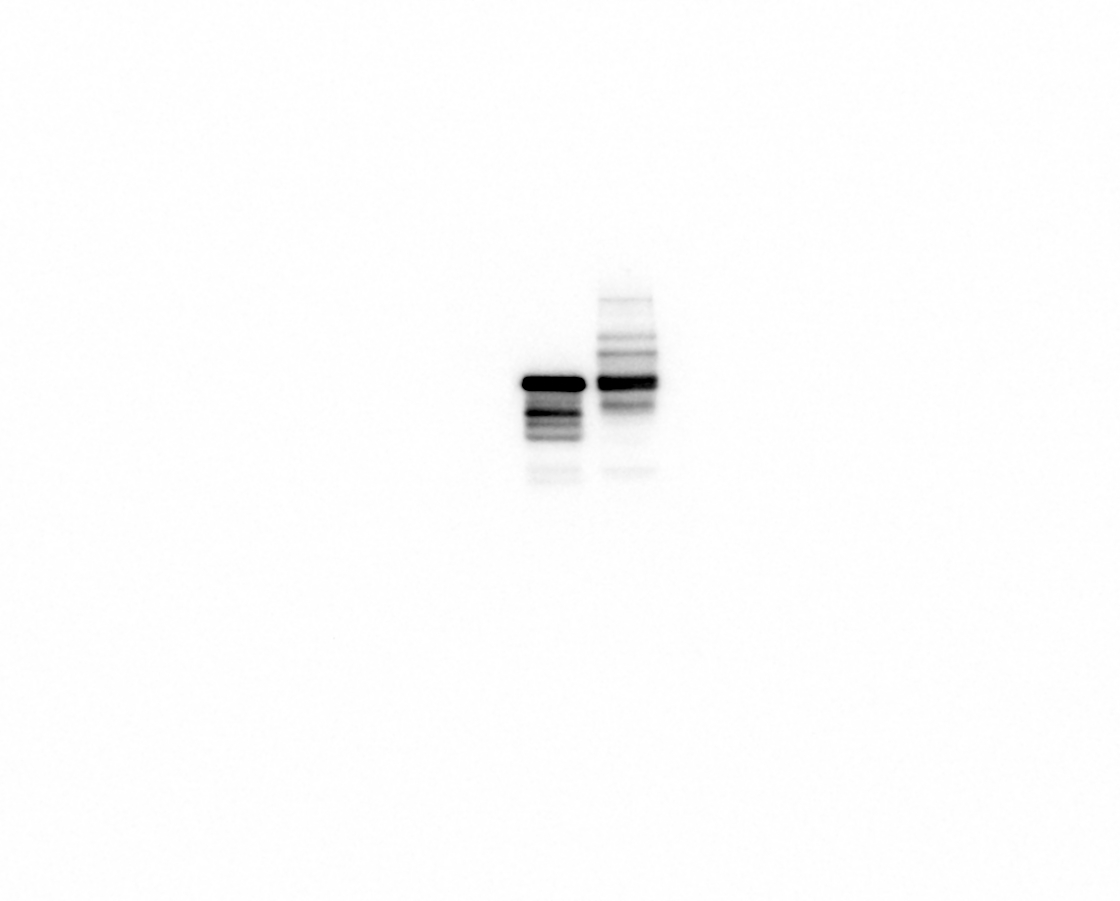

Supplement: Supplementary file 22 — Appendix Figures S1-S3 Source Data [file 44318_2025_516_MOESM22_ESM.zip › Appendix Figures/Appendix Figure S3/101323_SySy106008_40sExposure.jpg]

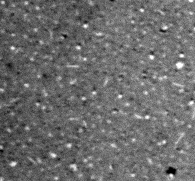

Supplement: Supplementary file 22 — Appendix Figures S1-S3 Source Data [file 44318_2025_516_MOESM22_ESM.zip › Appendix Figures/Appendix Figure S1/Panel B/EGFP at 0 min.tif]

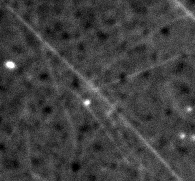

Supplement: Supplementary file 22 — Appendix Figures S1-S3 Source Data [file 44318_2025_516_MOESM22_ESM.zip › Appendix Figures/Appendix Figure S1/Panel B/actin at 45 min.tif]

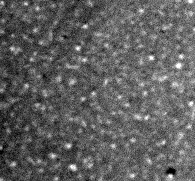

Supplement: Supplementary file 22 — Appendix Figures S1-S3 Source Data [file 44318_2025_516_MOESM22_ESM.zip › Appendix Figures/Appendix Figure S1/Panel B/EGFP at 45 min.tif]

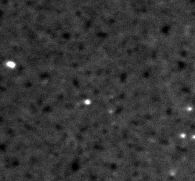

Supplement: Supplementary file 22 — Appendix Figures S1-S3 Source Data [file 44318_2025_516_MOESM22_ESM.zip › Appendix Figures/Appendix Figure S1/Panel B/actin at 0 min.tif]

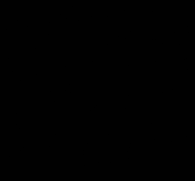

Supplement: Supplementary file 22 — Appendix Figures S1-S3 Source Data [file 44318_2025_516_MOESM22_ESM.zip › Appendix Figures/Appendix Figure S1/Panel A/t45_actin in reaction buffer.tif]

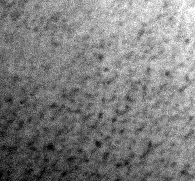

Supplement: Supplementary file 22 — Appendix Figures S1-S3 Source Data [file 44318_2025_516_MOESM22_ESM.zip › Appendix Figures/Appendix Figure S1/Panel A/t0_actin in polymerization buffer.tif]

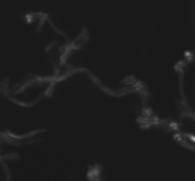

Supplement: Supplementary file 22 — Appendix Figures S1-S3 Source Data [file 44318_2025_516_MOESM22_ESM.zip › Appendix Figures/Appendix Figure S1/Panel A/t45_actin in polymerization buffer.tif]

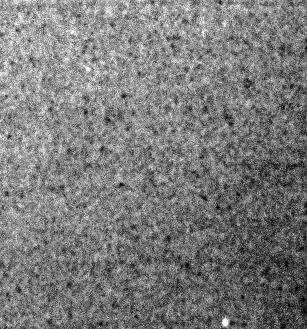

Supplement: Supplementary file 22 — Appendix Figures S1-S3 Source Data [file 44318_2025_516_MOESM22_ESM.zip › Appendix Figures/Appendix Figure S2/2 min/Actin.tif]

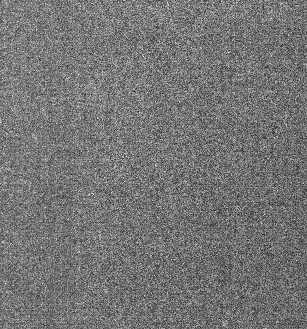

Supplement: Supplementary file 22 — Appendix Figures S1-S3 Source Data [file 44318_2025_516_MOESM22_ESM.zip › Appendix Figures/Appendix Figure S2/2 min/SVs.tif]

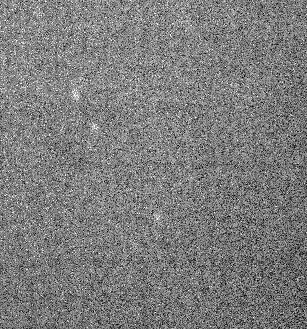

Supplement: Supplementary file 22 — Appendix Figures S1-S3 Source Data [file 44318_2025_516_MOESM22_ESM.zip › Appendix Figures/Appendix Figure S2/2 min/Syn1.tif]

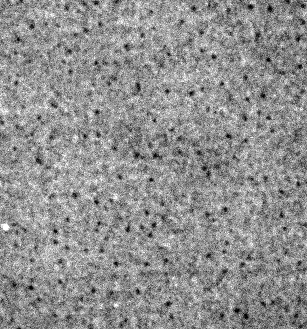

Supplement: Supplementary file 22 — Appendix Figures S1-S3 Source Data [file 44318_2025_516_MOESM22_ESM.zip › Appendix Figures/Appendix Figure S2/0 min/Actin.tif]

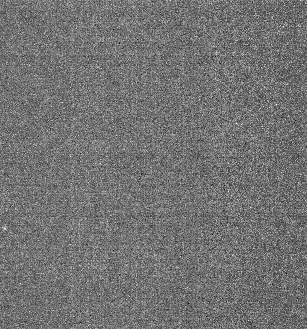

Supplement: Supplementary file 22 — Appendix Figures S1-S3 Source Data [file 44318_2025_516_MOESM22_ESM.zip › Appendix Figures/Appendix Figure S2/0 min/SVs.tif]

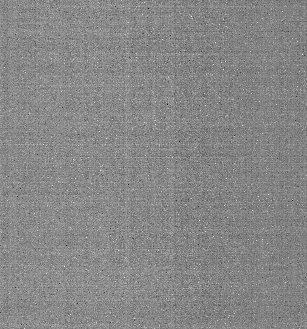

Supplement: Supplementary file 22 — Appendix Figures S1-S3 Source Data [file 44318_2025_516_MOESM22_ESM.zip › Appendix Figures/Appendix Figure S2/0 min/Syn1.tif]

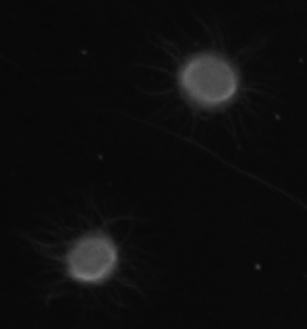

Supplement: Supplementary file 22 — Appendix Figures S1-S3 Source Data [file 44318_2025_516_MOESM22_ESM.zip › Appendix Figures/Appendix Figure S2/30 min/Actin-fire.tif]

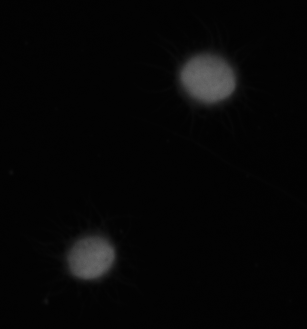

Supplement: Supplementary file 22 — Appendix Figures S1-S3 Source Data [file 44318_2025_516_MOESM22_ESM.zip › Appendix Figures/Appendix Figure S2/30 min/Syn1_fire.tif]

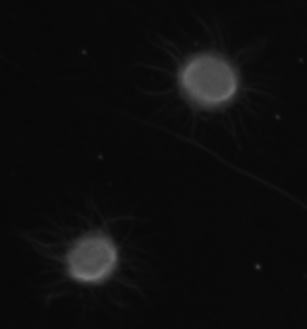

Supplement: Supplementary file 22 — Appendix Figures S1-S3 Source Data [file 44318_2025_516_MOESM22_ESM.zip › Appendix Figures/Appendix Figure S2/30 min/Actin.tif]

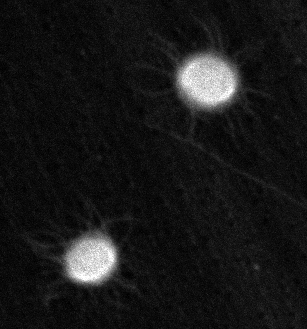

Supplement: Supplementary file 22 — Appendix Figures S1-S3 Source Data [file 44318_2025_516_MOESM22_ESM.zip › Appendix Figures/Appendix Figure S2/30 min/SVs.tif]

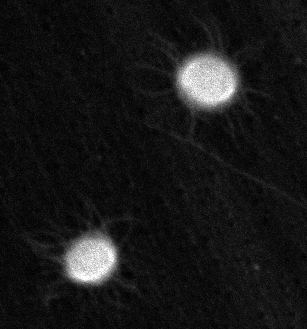

Supplement: Supplementary file 22 — Appendix Figures S1-S3 Source Data [file 44318_2025_516_MOESM22_ESM.zip › Appendix Figures/Appendix Figure S2/30 min/SVs-fire.tif]

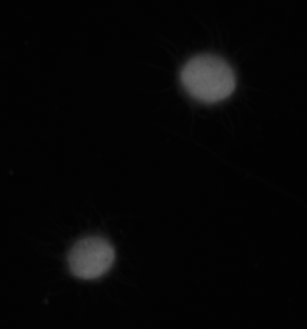

Supplement: Supplementary file 22 — Appendix Figures S1-S3 Source Data [file 44318_2025_516_MOESM22_ESM.zip › Appendix Figures/Appendix Figure S2/30 min/Syn1.tif]

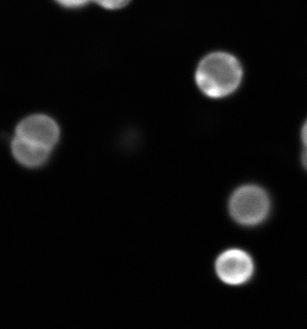

Supplement: Supplementary file 22 — Appendix Figures S1-S3 Source Data [file 44318_2025_516_MOESM22_ESM.zip › Appendix Figures/Appendix Figure S2/12 min/Actin.tif]

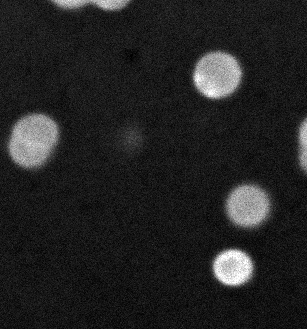

Supplement: Supplementary file 22 — Appendix Figures S1-S3 Source Data [file 44318_2025_516_MOESM22_ESM.zip › Appendix Figures/Appendix Figure S2/12 min/SVs.tif]

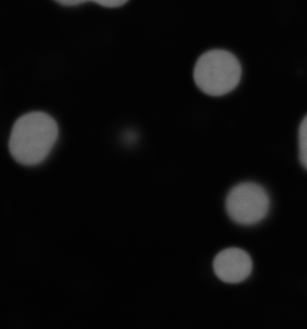

Supplement: Supplementary file 22 — Appendix Figures S1-S3 Source Data [file 44318_2025_516_MOESM22_ESM.zip › Appendix Figures/Appendix Figure S2/12 min/Syn1.tif]
