# Supplementary material for: Condensates of synaptic vesicles and synapsin-1 mediate actin sequestering and polymerization
Source: EMBO J. 2025 Aug 14;44(18):5112–48. doi: 10.1038/s44318-025-00516-y (PMC12436662; doi:10.1038/s44318-025-00516-y)
Supplement: Supplementary file 23 — Expanded View Figures [file 44318_2025_516_MOESM23_ESM.pdf]

Expanded View Figures

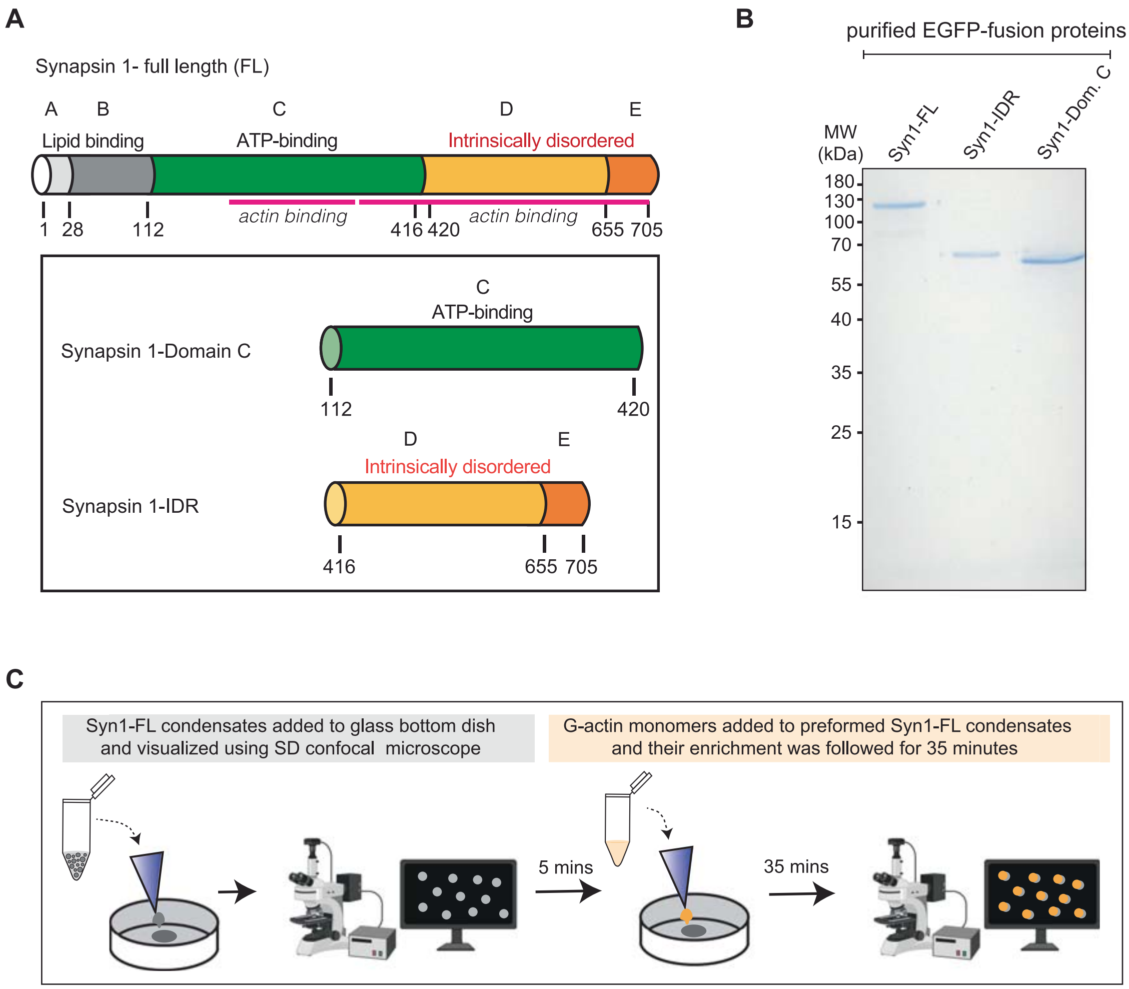

Figure EV1. Recombinant EGFP-synapsin-1 and domains used for in vitro reconstitutions.

(**A**) Schematic cartoon representing the domain organization of synapsin-1 (Syn1). Syn1 full length (FL), Domain C (Dom. C), and intrinsically disordered region (IDR) are depicted. Actin-binding regions are depicted with magenta (a.a. 222–370 and 371–705). (**B**) SDS-PAGE gel of the purified proteins employed for in vitro reconstitutions in this study: EGFP-Syn1-FL (102.235 kDa), EGFP-Syn1-IDR (57.621 kDa), and EGFP-Syn1-Dom. C (63.116 kDa). (**C**) Schematic illustration of the Syn1-actin reconstitution assay flow. Actin polymerization from Syn1 phases was examined by first preforming 6 μM EGFP-Syn1-FL condensates with 3% (w/v) PEG 8000 on a glass-bottom dish. After incubating for 5 min, when EGFP-Syn1-FL condensates became 3–4 μm in size, ATTO647-labeled G-actin monomers were added from the top into these preformed EGFP-Syn1-FL condensates such that the final concentration of actin and EGFP-Syn1-FL in the final reaction mix was 4 μM for both components. Source data are available online for this figure.

© The Author(s)

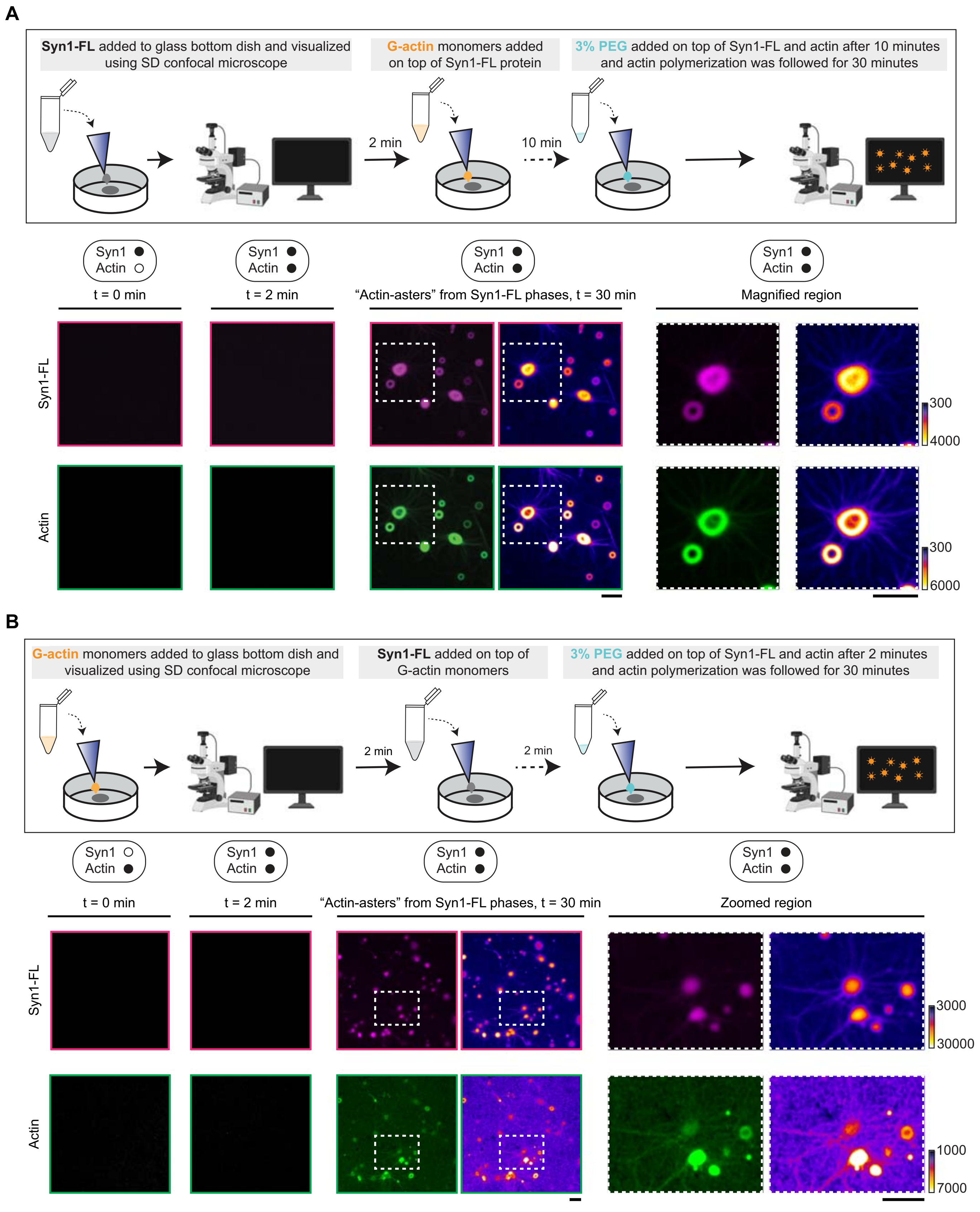
A
Syn1-FL added to glass bottom dish and visualized using SD confocal microscope
G-actin monomers added on top of Syn1-FL protein
3% PEG added on top of Syn1-FL and actin after 10 minutes and actin polymerization was followed for 30 minutes
2 min
10 min
Syn1
Actin
t = 0 min
t = 2 min
"Actin-asters" from Syn1-FL phases, t = 30 min
Magnified region
Syn1-FL
Actin
300
4000
300
6000
B
G-actin monomers added to glass bottom dish and visualized using SD confocal microscope
Syn1-FL added on top of G-actin monomers
3% PEG added on top of Syn1-FL and actin after 2 minutes and actin polymerization was followed for 30 minutes
2 min
2 min
Syn1
Actin
t = 0 min
t = 2 min
"Actin-asters" from Syn1-FL phases, t = 30 min
Zoomed region
Syn1-FL
Actin
3000
30000
1000
7000

 © The Author(s)

Figure EV2. Reconstitution of actin with synapsin-1 in two distinct reaction orders.

(**A**) Top: Schematic illustration showing the order-1 of the reconstitution assay. Actin polymerization from synapsin-1 phases was examined by first adding 4 μM synapsin-1 to a glass-bottom dish, followed by the addition of 4 μM ATTO647 G-actin monomers 2 min later. Subsequently, 3% (w/v) PEG 8000 was added on top of the reaction mix after 10 min, and actin polymerization was followed for 30 min. Bottom: Representative SD confocal microscopy images from the reconstitution of actin with synapsin 1 liquid phases in reaction buffer at $t = 0$, 2, and 30 min. Zoomed-in regions towards the right side show actin-asters. Images were acquired at 488 and 647 nm wavelengths for EGFP-Syn1-FL and actin, respectively. Scale bar, 5 μm. (**B**) Top: Schematic illustration showing the order-2 of the reconstitution assay. Actin polymerization from synapsin-1 phases was assessed by first adding 4 μM ATTO647 G-actin monomers to a glass-bottom dish. Two minutes later, 4 μM synapsin 1 was added on top of the reaction mix. Subsequently, 3% (w/v) PEG 8000 was added on top of the reaction mix after 2 min, and actin polymerization was followed for 30 min. Bottom: Representative confocal microscopy images from the reconstitution of actin with synapsin-1 liquid phases in reaction buffer at $t = 0$, 2, and 30 min. Zoomed-in regions towards the right side show actin-asters. Images were acquired at 488 and 647 nm wavelengths for EGFP-Syn1-FL and actin, respectively. Scale bar, 5 μm. Source data are available online for this figure.

© The Author(s)

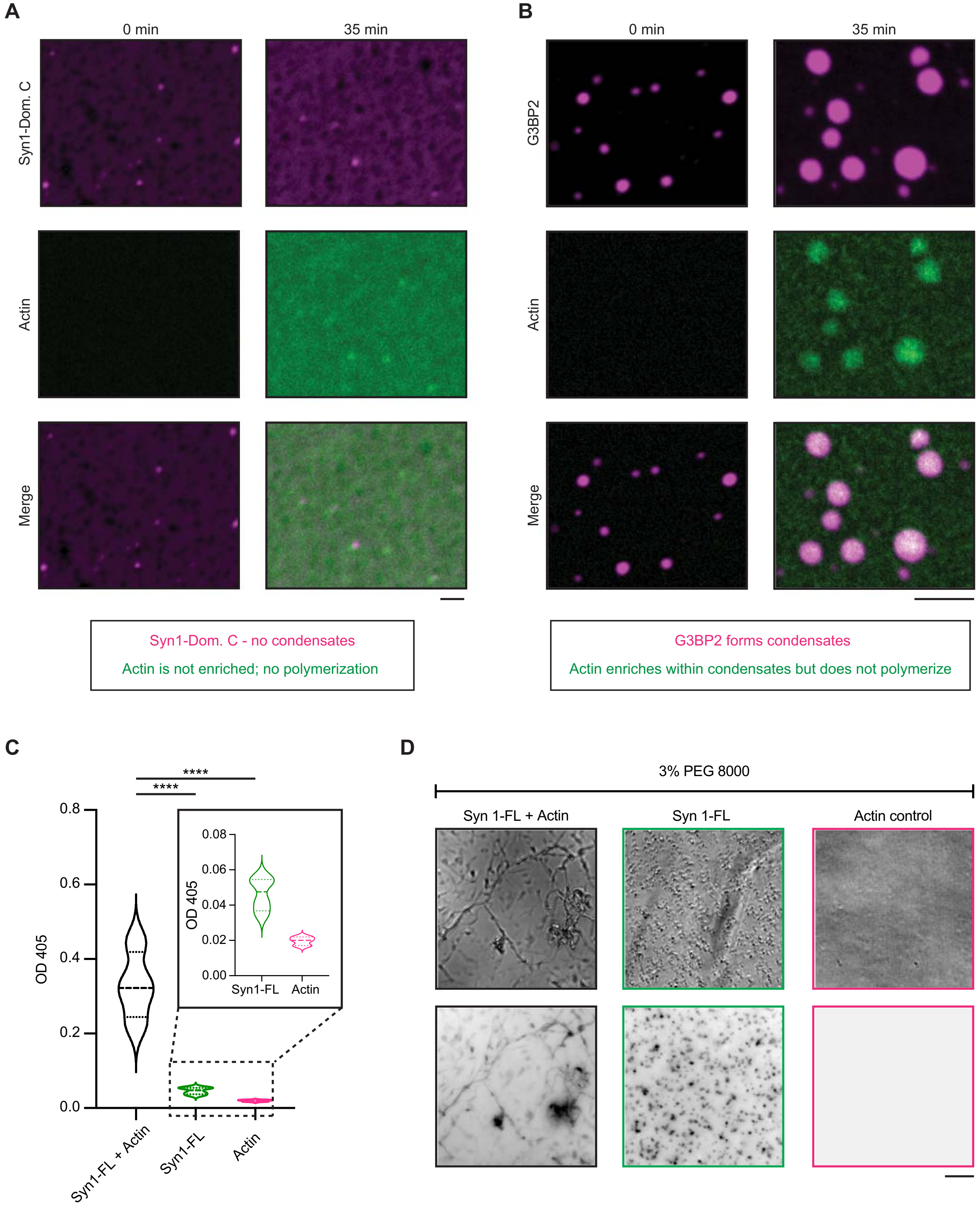
A
0 min
35 min
Syn1-Dom. C
Actin
Merge
Syn1-Dom. C - no condensates
Actin is not enriched; no polymerization
B
0 min
35 min
G3BP2
Actin
Merge
G3BP2 forms condensates
Actin enriches within condensates but does not polymerize
C
****
****
0.8
0.6
0.4
0.2
0.0
OD 405
Syn1-FL + Actin
Syn1-FL
Actin
0.08
0.06
0.04
0.02
0.00
OD 405
Syn1-FL
Actin
D
3% PEG 8000
Syn 1-FL + Actin
Syn 1-FL
Actin control

 © The Author(s)

Figure EV3. In vitro reconstitutions and turbidity measurements of Syn1 variants with actin.

(**A**) Representative confocal images from the in vitro reconstitution of EGFP-Syn1-Dom. C (4 µM, 3% (w/v) PEG 8000) with ATTO647-labeled G-actin monomers (4 µM) at $t = 0$ (left) and $t = 35$ min (right). Image acquisition for EGFP-Syn1-Dom. C and ATTO647 G-actin was carried out at excitation wavelengths 488 and 647 nm, respectively. Scale bar, 5 µm. (**B**) Representative confocal images from the in vitro reconstitution of EGFP-G3BP2 (4 µM, 3% PEG 8000) with ATTO647-labeled G-actin monomers (4 µM) at $t = 0$ (left) and $t = 35$ min (right). Images were acquired at excitation wavelengths 488 and 647 nm for EGFP-G3BP2 and ATTO647 G-actin, respectively. Scale bar, 5 µm. (**C**) Quantification of the turbidity assay. Plot comparing the turbidity measurements for EGFP-Syn1-FL with actin, EGFP-Syn1-FL alone, and actin alone in the presence of 3% (w/v) PEG 8000. Actin polymerization was assessed as an increase in optical density after a 48 h incubation period. Actin used for the assay was Mg^{2+}-exchanged and supplemented with 0.5 mM ATP. Data shown here is quantified from four independent experiments ($N = 4$). The p values are: 8.53×10^{-4} for Syn1-FL+Actin Vs Syn1-FL and 1.13×10^{-4} for Syn1-FL+Actin Vs Syn1-FL Vs Actin; ****$p < 0.0001$; one-way ANOVA test. (**D**) Top: representative brightfield images of reaction mixes after turbidity assay from (**A**). Bottom: epifluorescence images of the same regions at 488 nm excitation wavelength. Scale bar, 50 µm. Source data are available online for this figure.

© The Author(s)

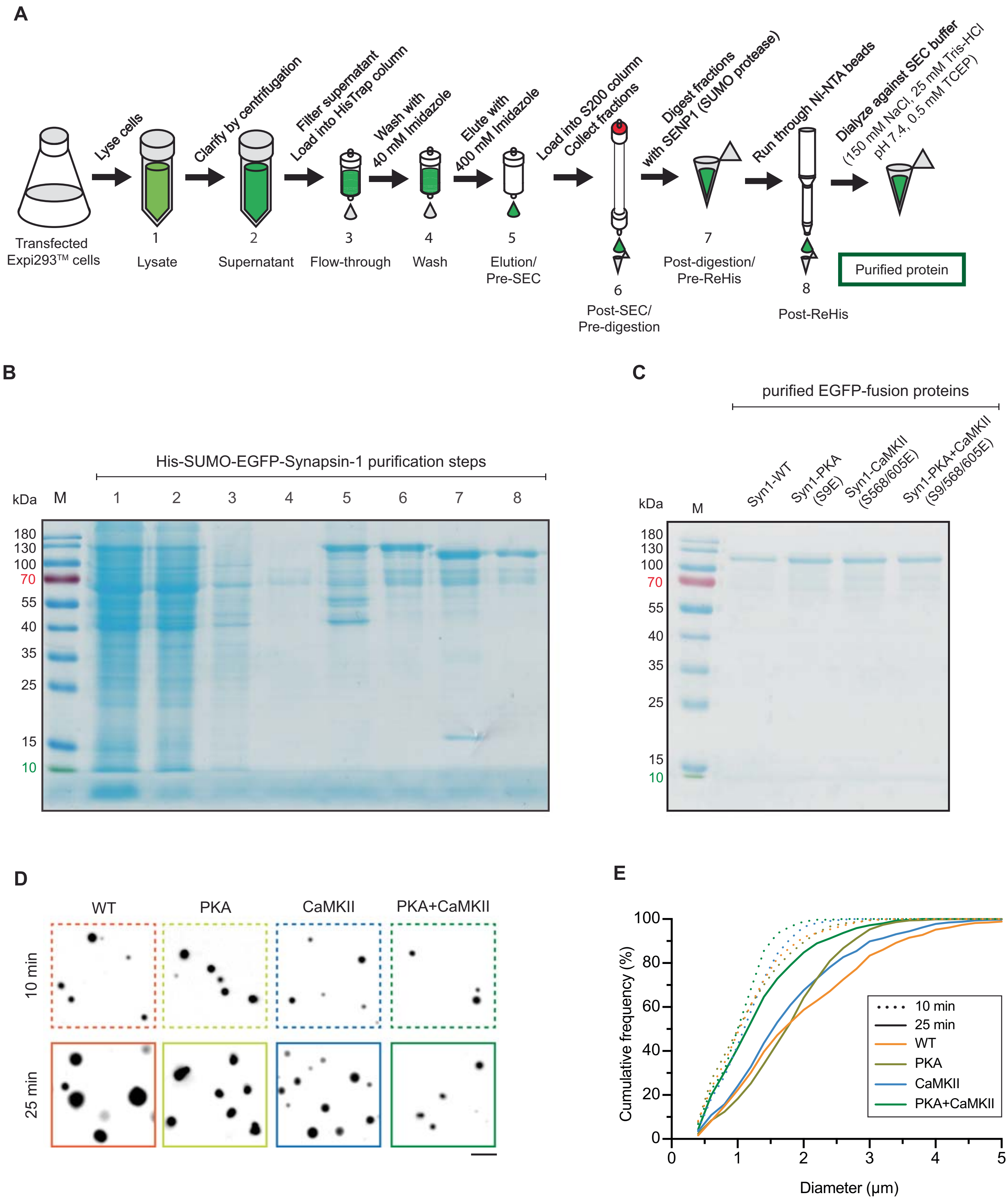
A
Lyse cells
Clarify by centrifugation
Filter supernatant
Load into HisTrap column
Wash with
40 mM Imidazole
Elute with
400 mM Imidazole
Load into S200 column
Collect fractions
Digest fractions
with SENP1 (SUMO protease)
Run through Ni-NTA beads
Dialyze against SEC buffer
(150 mM NaCl, 25 mM Tris-HCl
pH 7.4, 0.5 mM TCEP)
Transfected Expi293TM cells
1 Lysate
2 Supernatant
3 Flow-through
4 Wash
5 Elution/ Pre-SEC
6 Post-SEC/ Pre-digestion
7 Post-digestion/ Pre-ReHis
8 Post-ReHis
Purified protein
B
His-SUMO-EGFP-Synapsin-1 purification steps
kDa M 1 2 3 4 5 6 7 8
180 130 100 70 55 40 35 25 15 10
C
purified EGFP-fusion proteins
Syn1-WT
Syn1-PKA (S9E)
Syn1-CaMKII (S568/605E)
Syn1-PKA+CaMKII (S9/568/605E)
kDa M
180 130 100 70 55 40 35 25 15 10
D
WT PKA CaMKII PKA+CaMKII
10 min
25 min
E
Cumulative frequency (%)
Diameter (µm)
10 min
25 min
WT
PKA
CaMKII
PKA+CaMKII

Figure EV4. Recombinant synapsin 1 phosphomimetics used for in vitro reconstitutions.

(**A**) Schematic cartoon depicting the purification steps followed to obtain synapsin 1 (Syn1) from transfected Expi293™ cells expressing His-SUMO-EGFP-Syn1. This same pipeline is used for all Syn1 variants. (**B**) Exemplary SDS-PAGE gel for His-SUMO-EGFP-Syn1-CaMKII (S568/605E) construct, showing the fractions from each purification step from 1 to 8 in (**A**). (**C**) SDS-PAGE gel of the final purified EGFP-Syn1 versions employed for in vitro reconstitutions in this study: EGFP-Syn1 WT, EGFP-Syn1-PKA (S9E), EGFP-Syn1-CaMKII (S568/605E), and EGFP-Syn1-PKA+CaMKII (S9/568/605E). All proteins weigh 102.235 kDa. (**D**) Representative images of EGFP-Syn1 WT, EGFP-Syn1-PKA (S9E), EGFP-Syn1-CaMKII (S568/605E), and EGFP-Syn1-PKA+CaMKII (S9/568/605E) when reconstituted in SEC buffer and in the presence of 3% PEG 8000 at $t = 10$ and $t = 25$ min. Images were acquired using a spinning-disk confocal microscope, employing the 488 nm wavelength for EGFP-Syn1. Scale bar, 5 µm.
(**E**) Cumulative frequency indicating the size distribution of at $t = 0$ (full line) and $t = 45$ min (dotted line); color code as in (D). Data from three independent reconstitutions for each condition. Source data are available online for this figure.

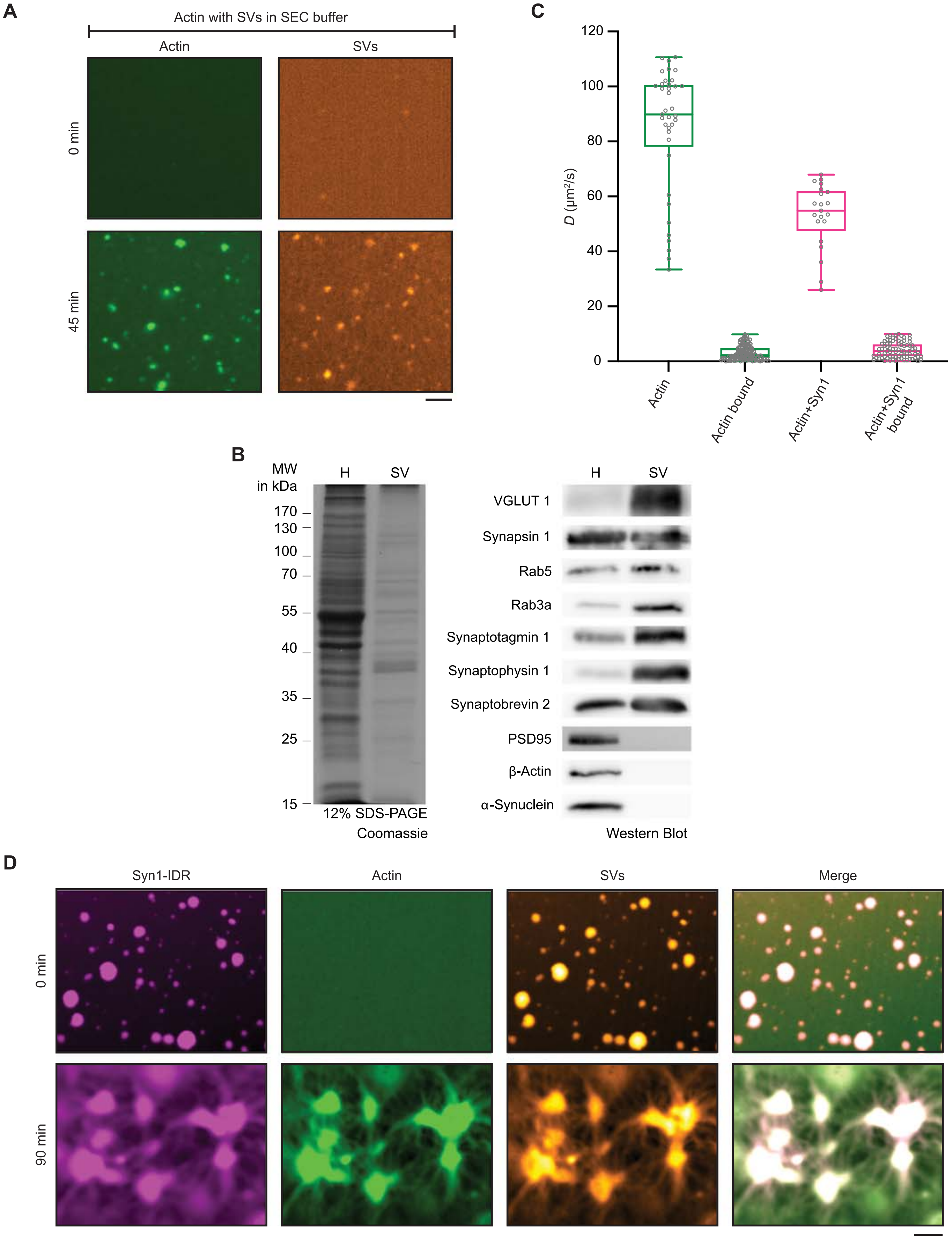
A
Actin with SVs in SEC buffer
Actin
SVs
0 min
45 min
C
D (μm²/s)
120
100
80
60
40
20
0
Actin
Actin bound
Actin+Syn1
Actin+Syn1 bound
B
MW in kDa
H
SV
170
130
100
70
55
40
35
25
15
12% SDS-PAGE
Coomassie
VGLUT 1
Synapsin 1
Rab5
Rab3a
Synaptotagmin 1
Synaptophysin 1
Synaptobrevin 2
PSD95
β-Actin
α-Synuclein
Western Blot
D
Syn1-IDR
Actin
SVs
Merge
0 min
90 min

 © The Author(s)

Figure EV5. Reconstitution of actin with synapsin-1 and native SVs.

(**A**) Representative images for actin reconstitution in the presence of natively purified 3 nM SVs labeled with FM4-64 (1.65 μM final concentration), 3% PEG 8000 in reaction buffer at $t = 0$ and $t = 45$ min. Images were acquired using a spinning-disk confocal microscope at 561 and 647 nm for SVs and actin, respectively. Scale bar, 5 μm. (**B**) Quality control of synaptic vesicles. Left: Coomassie-stained 12% SDS-PAGE of fractions, brain homogenate (H) and final synaptic vesicle fraction (SV), from the synaptic vesicle isolation procedure from native rat brain (5 μg per lane). Right: Western blot of brain homogenate and final SV fraction detecting classical protein markers of SV enrichment and absence of proteins regarded as contaminants. (**C**) Diffusion coefficients of actin bound to SVs immobilized on a functional surface as described in Perego et al, 2020. Data from three independent replicates ($N = 3$). The box stretches from the 25th to the 75th percentile, the dots represent individual data points, the central line shows the median, and the whiskers represent the min and max values. Green boxes, diffusion of actin alone; magenta boxes, diffusion of actin in the presence of EGFP-synapsin 1. (**D**) Reconstitution of actin with synapsin-1 IDR-SV phases and actin-asters. Representative confocal images of the reconstituted EGFP-Syn1-IDR (4 μM, 3% PEG 8000) and SVs (3 nM, labeled with FM4-64, 1.65 μM) condensates after adding ATTO647-labeled G-actin monomers (4 μM) at $t = 0$ (top) and 90 min (bottom). Excitation at 488 nm for EGFP-synapsin 1-IDR, 560 nm for SVs labeled with FM4-64 and 647 nm for ATTO647 G-actin. Scale bar, 5 μm. Source data are available online for this figure.

© The Author(s)

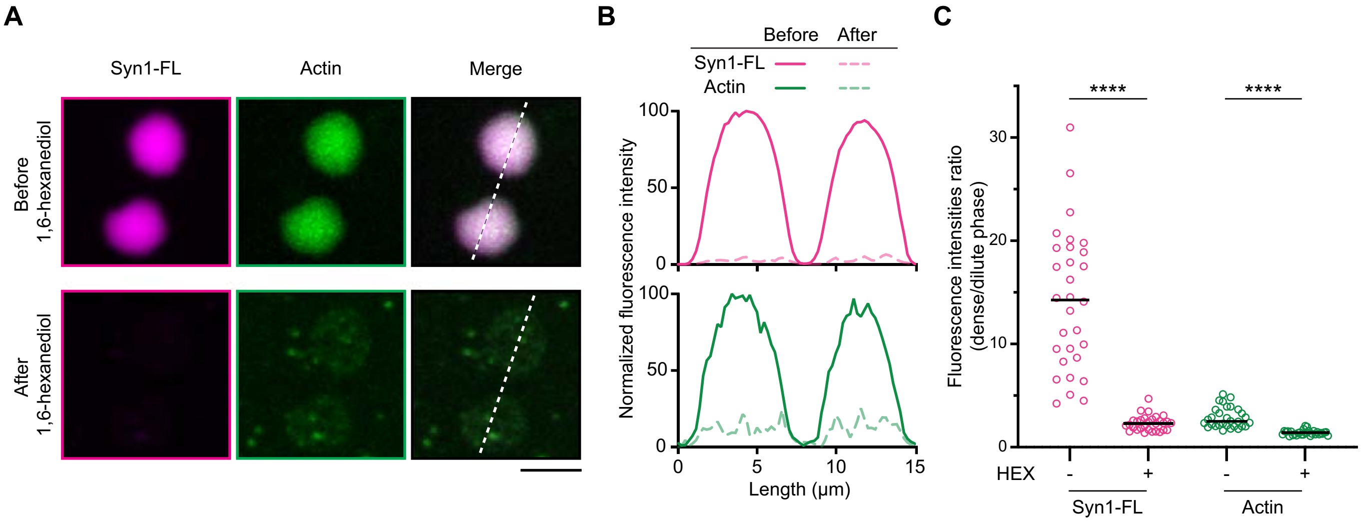

Figure EV6. 1,6-Hexanediol disperses EGFP-synapsin 1:actin condensates lacking an apparent actin polymerization.

(**A**) Representative confocal images from the in vitro reconstituted EGFP-synapsin-1:actin:GUV assemblies before and after 1,6-hexanediol treatment. The dashed line represents the line used for plotting the intensity profile. Scale bar, 5 µm. (**B**) Fluorescence intensity profiles of EGFP-synapsin-1 and actin along the dashed line from (**A**). Solid lines, fluorescence intensity before, and dashed lines, after treatment with 1,6-hexanediol. (**C**) Quantification of synapsin-1 and actin partitioning in EGFP-synapsin-1: actin:GUV assemblies before and after 1,6-hexanediol treatment. Data from three independent reconstitutions, 30 condensates analyzed for each condition. The p values are 6.76×10^{-17} for Syn1-FL before vs. after treatment and 6.31×10^{-15} for actin before vs after treatment. ****$p < 0.0001$; Mann–Whitney U-test (two-tailed, non-parametric). Source data are available online for this figure.

 © The Author(s)

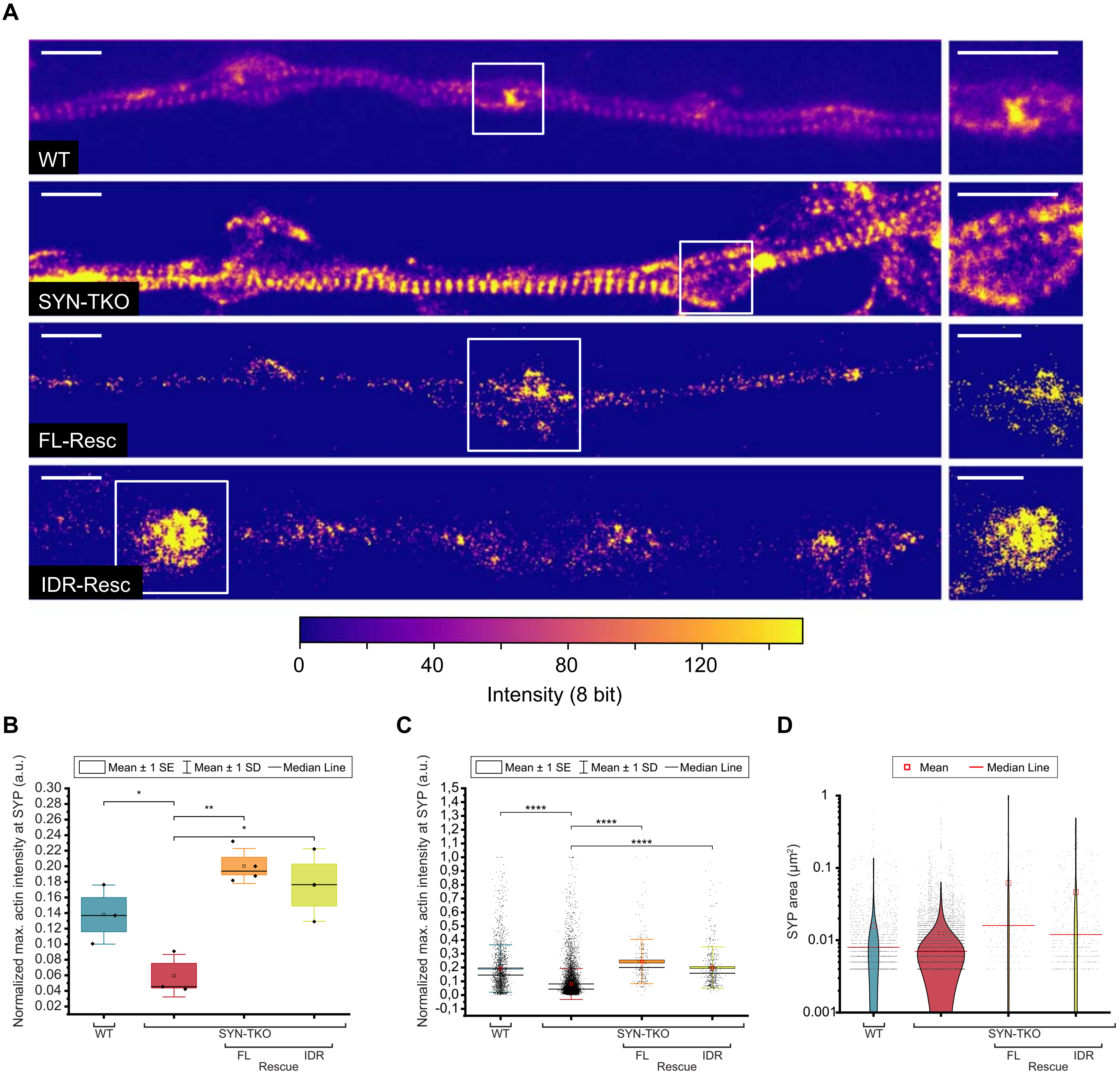

Figure EV7. Synaptic vesicle condensates are necessary for concentrating actin at the presynaptic boutons.

(**A**) Primary hippocampal neurons in culture (DIV 14) stained for actin (phalloidin-AberriorStarRed); images as in Fig. 8, but here shown with the same intensity scaling. Scale bars, 1 µm. (**B**) Actin enrichment in synaptic vesicle cohorts is defined as a normalized intensity signal of actin channel within regions positive for synaptophysin (wild-type and synapsin triple knockout) or both synaptophysin and EGFP (in rescue experiments). Data from at least three independent neuronal preparations (for WT: $N = 3$; $n = 3$; for SynTKO: $N = 3$; $n = 3$; for SynTKO+FL: $N = 4$; $n = 4$; for SynTKO+IDR: $N = 3$; $n = 3$); data points represent median maximum actin intensity per neuronal preparation; the box represents mean ± 1.0 SE, whiskers represent mean ± 1.0 SD, and the central line represent median two sample *t*-test (equal variance not assumed). WT-SynTKO $p = 0.0049$, *; SynTKO-SynTKO+FL $p = 0.0021$, **; SynTKO-SynTKO+IDR $p = 0.030$, *. (**C**) Actin enrichment in synaptic vesicle cohorts is defined as a normalized intensity signal of actin channel within regions positive for synaptophysin (wild-type and synapsin triple knockout) or both synaptophysin and EGFP (in rescue experiments). Data from three independent neuronal preparations (for WT: $N = 3$; $n = 1592$; for SynTKO: $N = 3$; $n = 7096$; for SynTKO+FL: $N = 4$; $n = 209$; for SynTKO +IDR: $N = 3$; $n = 460$); data points represent maximum actin intensity per region positive for synaptophysin. The box represents mean ± 1.0 SE, and whiskers represent mean ± 1.0 SD, and the central line represents median. Mann–Whitney test; WT-SynTKO, $p = 3.06$ x 10^(-251); SynTKO-SynTKO+FL, $p = 2.12 \times 10^{-79}$; SynTKO-SynTKO +IDR, $p = 1.96 \times 10^{-135}$; ****$p < 0.0001$. (**D**) Area of synaptophysin-positive regions in different conditions. Note in SynTKO neurons the presence of regions of varying size, particularly the smaller ones, presumably a consequence of dispersed SVs into smaller cohorts and/or individual vesicles. The red square represents mean values, and the central line shows the median. Data from at least three independent replicates (for WT: $N = 3$; $n = 1592$; for SynTKO: $N = 3$; $n = 7096$; for SynTKO+FL: $N = 4$; $n = 209$; for SynTKO+IDR: $N = 3$; $n = 460$). Source data are available online for this figure.

© The Author(s)
